# Pleiotropic Effects of Metformin on the Chemotherapy Response of HPV‐Positive Cancer Cells

**DOI:** 10.1002/jmv.70434

**Published:** 2025-06-16

**Authors:** Alicia Avenhaus, Bianca J. Kuhn, Milica Velimirović, Tobias D. Strobel, Julia Bulkescher, Claudia Lohrey, Jeroen Krijgsveld, Felix Hoppe‐Seyler, Karin Hoppe‐Seyler

**Affiliations:** ^1^ German Cancer Research Center (DKFZ) Molecular Therapy of Virus‐Associated Cancers Heidelberg Germany; ^2^ Faculty of Biosciences Heidelberg University Heidelberg Germany; ^3^ German Cancer Research Center (DKFZ) Division of Proteomics of Stem Cells and Cancer Heidelberg Germany; ^4^ The Broad Institute of MIT and Harvard Cambridge MA USA; ^5^ Medical Faculty Heidelberg University Heidelberg Germany

**Keywords:** cervical cancer, chemotherapy, human papillomavirus (HPV), Metformin, oncogenesis, oncoproteins

## Abstract

Improved treatment strategies for HPV‐positive cancers are urgently required. The viral E6/E7 oncoproteins are essential for the proliferation of HPV‐positive cancer cells and considered attractive therapeutic targets. Metformin is proposed to be repurposed for cancer therapy, but this is under controversial debate. We previously demonstrated that E6/E7 expression and the proliferation of HPV‐positive cancer cells are repressed by Metformin. Here, we explore the effects of Metformin on the phenotype of HPV‐positive cancer cells in detail, either applied as monotreatment or in combination with chemotherapeutic agents. We provide evidence that the downregulation of E6/E7 is not the primary mechanism underlying Metformin's growth‐inhibitory effect in HPV‐positive cancer cells. Specifically, compared to targeted E6/E7 repression by RNA interference (RNAi), Metformin treatment differently altered the expression of growth regulatory proteins, exerted different effects on the cell cycle, and was able to suppress growth even in the presence of E6/E7. Furthermore, we found that cancer cells pre‐treated with Metformin become resistant to senescence induction by the pro‐senescent chemotherapeutic agent Etoposide, likely as a secondary effect of Metformin‐induced growth inhibition. Finally, depending on experimental conditions, we uncover divergent, even opposing, effects on the proliferation of HPV‐positive cancer cells when Metformin is combined with Cisplatin, with p53 playing a key role in these processes. Collectively, our results show that Metformin exerts complex effects on the phenotype of HPV‐positive cancer cells, which are critically influenced by experimental conditions. Our findings may also explain the discrepant results in the literature, reporting agonistic or antagonistic effects upon combining Metformin with Cisplatin.

## 1 Introduction

High‐risk human papillomaviruses (HPVs), such as HPV16 and HPV18, contribute to almost 5% of the global cancer incidence by causing several anogenital cancers, including cervical cancer, and a substantial portion of head and neck cancers [1, 2]. While HPV vaccination programs have significantly reduced cervical cancer incidence in certain regions, HPV continues to cause approximately 600 000 new cervical cancer cases and over 300 000 deaths from cervical cancer each year [3]. Current treatment strategies for cervical cancer depend on the stage and extent of cancer progression [4], with Cisplatin‐based chemoradiation being a standard treatment for locally advanced and recurrent disease [5]. However, 5‐year overall survival for locally advanced cervical cancer remains 65%, and up to 40% of patients develop recurrent disease [6], highlighting the urgent need for improved therapeutic approaches. The induction of apoptosis and/or the induction of senescence in tumor cells are two major mechanisms by which chemotherapeutic agents exert their anti‐cancer effects [7, 8, 9, 10, 11, 12]. Therefore, novel strategies that enhance the pro‐apoptotic or pro‐senescent effects of chemotherapy (CT) could provide more effective therapeutic outcomes.

Metformin, a biguanide class drug widely used to treat type 2 diabetes mellitus (T2DM), may possess the potential to be repurposed for cancer therapy, since several studies reported promising anti‐cancer effects In Vitro and In Vivo [13, 14, 15, 16, 17]. This concept, however, is controversially discussed, and a number of clinical studies investigating the anti‐cancer potential of Metformin led to rather disappointing results [17]. Conflicting data have also been reported for cervical cancer with studies showing that Metformin reduced the risk of cervical cancer development [18, 19] and decreased cervical cancer‐linked mortality [20] in T2DM patients, whereas no beneficial effects were observed in other reports [21, 22]. Of further note, a broad range of studies investigated the effects of Metformin not only as a single agent in cancer treatment, but also as an adjuvant to CT. This combination has been suggested to enhance the anti‐cancer efficacy of CT in several investigations, yet, there are also studies reporting no improvements or even decreased CT efficacy when combined with Metformin [17, 23, 24, 25]. In addition, the mechanisms by which Metformin could influence the response of cancer cells to CT remain poorly understood.

HPV‐driven oncogenesis is mediated by the viral E6 and E7 oncoproteins, which inactivate p53 and retinoblastoma protein (pRb) tumor suppressor pathways, promoting deregulated cellular proliferation [26]. Inhibition of viral *E6/E7* oncogene expression results in rapid induction of cellular senescence [27, 28, 29], an irreversible growth arrest [30], in HPV‐positive cancer cells, underscoring the potential of E6 and E7 to serve as therapeutic targets [26, 31, 32]. Interestingly, we previously found that Metformin inhibits viral *E6/E7* oncogene expression in HPV‐positive cancer cells at both transcript and protein level [33]. However, despite effective E6/E7 repression, Metformin treatment does not result in senescence and only induces a reversible growth arrest, as cells resume growth upon Metformin removal. In addition, we obtained evidence that Metformin may also confer protection against Etoposide‐induced senescence in HPV‐positive cancer cells [33].

In the present work, we uncover pronounced differences in the phenotypic response of cervical cancer cells upon RNAi‐mediated or Metformin‐induced E6/E7 repression. We show that Metformin blocks their proliferation by differentially affecting cell cycle regulation independent of its ability to downregulate E6/E7 expression. Moreover, we analyze the effects of Metformin on the CT response of cervical cancer cells. We show that, depending on specific treatment conditions, Metformin treatment can have different short‐ and long‐term effects when combined with pro‐senescent or pro‐apoptotic CT and provide insights into the underlying cellular mechanisms.

## 2 Materials and Methods

### 2.1 Cell Culture and Treatment Conditions

HPV18‐positive HeLa (RRID: CVCL_0030) and HPV16‐positive SiHa (RRID: CVCL_0032) cervical cancer cell lines, as well as HPV‐negative HCT116 (RRID: CVCL_0291) colon cancer cells, MCF‐7 (RRID: CVCL_0031) breast cancer cells, and U2OS (RRID: CVCL_0042) osteosarcoma cells were obtained from the tumor bank of the German Cancer Research Center (DKFZ) in Heidelberg. SiHa‐mKate2 cells, which stably express a nuclear‐restricted form of the mKate2 fluorescent protein, were generated as previously described [11]. Cells were cultured at 37°C, 5% CO_2_, and 21% O_2_ in Dulbecco's minimal essential medium (DMEM) (HeLa, SiHa, MCF‐7, U2OS, SiHa‐mKate2) or McCoy's 5 A medium (HCT116) (Gibco, Thermo Fisher Scientific) containing 1 g/L (5.5 mM) glucose, if not stated otherwise. Media were additionally supplemented with 10% fetal bovine serum (PAN‐Biotech), 2 mM L‐glutamine, 100 U/mL penicillin, and 100 µg/mL streptomycin (all from Sigma Aldrich). All cell lines were authenticated through single‐nucleotide polymorphism profiling (Multiplexion GmbH) within the last 2 years and were confirmed negative for mycoplasma contamination.

Metformin (Enzo Life Sciences) dissolved in DMEM, Nutlin‐3, Nocodazole (both from Cayman Chemical Company), and Etoposide (Enzo Life Sciences), all dissolved in dimethyl sulfoxide (DMSO), and Cisplatin (Sigma Aldrich) dissolved in 0.9% NaCl/H_2_O were used for treatment. Final concentrations as well as specific treatment schedules are described in the figures and their corresponding legends.

### 2.2 RNAi

Silencer Select small interfering RNAs (siRNAs) (Life Technologies, Thermo Fisher Scientific) were transfected at a final concentration of 10 nM using Lipofectamine RNAiMAX (Invitrogen, Thermo Fisher Scientific) or DharmaFECT I (Dharmacon, Horizon Discovery), as per the manufacturer's protocol. For targeting HPV18 E6/E7, HPV16 E6/E7, or E6AP, equimolar pools of three different siRNAs (referred to in the text as si18E6/E7, si16E6/E7, or siE6AP) were used and have been characterized before [34, 35, 36, 37]. Other siRNA target sequences employed were: sip53: 5′‐GACUCCAGUGGUAAUCUAC‐3′ [38], siBID: 5′‐CUUGCUCCGUGAUGUCUUU‐3′ [39]; and control siRNAs: siCtrl1: 5′‐CAGUCGCGUUUGCGACUGG‐3′ or siNeg: 5′‐UACGACCGGUCUAUCGUAG‐3′, both containing at least four mismatches to all known human genes [35, 40, 41].

### 2.3 Immunoblotting

Protein extraction and immunoblot analyses were performed as described previously [35, 37, 39]. The following primary antibodies were used: anti‐HPV18 E6 (AVC 399) and anti‐HPV16 E6 (AVC 843, kind gifts from Dr. Johannes Schweizer, Arbor Vita Corporation, Fremont, CA, USA); anti‐HPV18 E7 (E7C) [42]; anti‐HPV16 E7 (NM2, kind gift from Dr. Martin Müller, German Cancer Research Center, Heidelberg, Germany); anti‐B‐MYB (LX015.1, from Dr. Roger Watson) [43]; anti‐BID (#2002), anti‐cleaved Caspase‐9 (#7237), anti‐cleaved poly(ADP‐ribose) polymerase (PARP) (#9546), anti‐FOXM1 (D3F2B, #20549), anti‐acetyl‐p53 (K382) (#2525), anti‐phospho‐p53 (S15) (#9284), anti‐p130 (D9T7M, #13610), anti‐pRb (#9309), anti‐phospho‐pRb (S807/811) (#9308), all from Cell Signaling Technology; anti‐CKS1 (36‐6800), from Invitrogen, Thermo Fisher Scientific; anti‐β‐Actin (C4, sc‐47778), anti‐CDC2 (B‐6, sc‐8395), anti‐CDK2 (D‐12, sc‐6248), anti‐Cyclin A (H‐432, sc‐751), anti‐Cyclin B2 (A‐2, sc‐28303), anti‐Cyclin D1 (DCS‐6, sc‐20044), anti‐E2F1 (KH95, sc‐251), anti‐glyceraldehyde 3‐phosphate dehydrogenase (GAPDH) (FL‐335, sc‐25778), anti‐p21 (SX118, sc‐53870), anti‐p53 (DO‐1, sc‐126), anti‐PCNA (PC10, sc‐56), anti‐PLK1 (F‐8, sc‐17783), anti‐RRM1 (R1 [A‐10], sc‐377415), anti‐RRM2 (R2 [A‐5], sc‐398294), all from Santa Cruz Biotechnology; anti‐Cyclin B1 (05‐373, clone GNS3 [8A5D12]), from Upstate; anti‐E6AP (E8655, clone E6AP‐300), from Sigma‐Aldrich.

The following horseradish peroxidase (HRP)‐conjugated secondary antibodies were used: anti‐chicken IgY‐HRP (sc‐2428) from Santa Cruz Biotechnology; anti‐mouse IgG (H + L), HRP conjugate (W4021), and anti‐rabbit IgG (H + L), HRP conjugate (W4011) from Promega.

Immunoblots were visualized using the enhanced chemiluminescence (ECL) reagents Western Bright Sirius (Advansta), or Amersham ECL Prime (Cytiva), and the Fusion SL Detection System (Vilber Lourmat). All immunoblot analyses were conducted at least three times, with consistent results. Densitometric quantification of immunoblots was carried out using Fiji/ImageJ 1.52e [44] following the pixel density quantification method described by Hossein Davarinejad [45], and is summarized in Table S1.

### 2.4 RNA Analyses

RNA extraction and subsequent quantitative reverse transcription‐polymerase chain reaction (qRT‐PCR) were performed as described before [39]. Primers with the following sequences were used: TP53 for: 5′‐ CTGAGGTTGGCTCTGACTGT‐3′; TP53 rev: 5′‐ CAAAGCTGTTCCGTCCCAGT‐3′; TMBIM6 for: 5′‐GTGGTCATGTGTGGCTTCGT‐3′; TMBIM6 rev: 5′‐GGAAAGGCTGGATGGTCACT‐3′. qRT‐PCR analyses were performed in three independent biological replicates. The comparative C_t_ ($2^{-∆∆C_{t}}$) method [46] was used to quantify relative mRNA levels. C_t_ values were normalized to the internal reference gene *TMBIM6*.

### 2.5 Cell Cycle Analyses

For cell cycle analyses, cells were harvested and processed as described previously [37]. Cell cycle distribution was evaluated using the BD LSRFortessa (BD Biosciences) and the BD FACS DIVA Software v8.0.1. Data analyses were performed using FlowJo v10.8.1 Software (BD Life Sciences) and the Dean–Jett–Fox model was applied to quantify the number of cells in individual cell cycle phases [47]. Cell cycle analyses were performed in three independent biological replicates.

### 2.6 Live‐Cell Imaging

SiHa‐mKate2 cells were seeded in 96‐well plates at a density of 3000 cells per well. Imaging was conducted with the Incucyte S3 device (Sartorius) every 24 h using 10× magnification. Cell proliferation was assessed by counting viable cells (red objects) using the Incucyte software (v2021C) and red object counts were normalized to the counts at the start of treatment (time point 0, set to 1). Live‐cell imaging experiments were conducted three times, with consistent results.

### 2.7 Terminal Deoxynucleotidyl Transferase‐Mediated UTP End Labeling (TUNEL) Assays

TUNEL assays were carried out using the “In Situ Cell Death Detection Kit” (Roche) according to the manufacturer's protocol. Cell imaging was performed using a Zeiss Cell Observer Microscope (Zeiss) with a 20× objective. Quantifications were conducted with a Fiji/ImageJ [44] macro generously provided by Dr. Damir Krunic from the Light Microscopy Core Facility of the German Cancer Research Center [11, 37, 39]. TUNEL‐positive cells and the total number of cells (identified through 4′,6‐diamidino‐2‐phenylindole [DAPI] staining, Roche) were quantified from a minimum of five images per condition. TUNEL assays were performed in three independent biological replicates.

### 2.8 Senescence and Colony Formation Assays (CFAs)

Following treatment, cells were split and reseeded in drug‐free medium. For senescence assays, cells were fixed with 2% formaldehyde and 0.2% glutaraldehyde in phosphate‐buffered saline and stained using a solution containing 1 mg/mL X‐Gal, 5 mM K_3_[Fe(CN)_6_], 5 mM K_4_[Fe(CN)_6_], 40 mM citric acid, 150 mM NaCl, and 2 mM MgCl_2_ at pH 6.0 and 37°C for 24 h. Images were captured with the EVOSxl Core Cell Imaging System (Invitrogen, Thermo Fisher Scientific) using 20× magnification. All senescence experiments were conducted at least three times, with consistent results. For CFAs, cells were fixed with formaldehyde and stained with crystal violet. Colony areas from three independent CFA experiments were quantified with Fiji/ImageJ 1.52e [44] using the ColonyArea plugin [48] and are shown relative to controls as described in the corresponding figure legends.

### 2.9 Sample Preparation and Data Analysis for Tandem Mass Tag (TMT)‐Mass Spectrometry (MS)‐Based Proteomics

For proteome analyses, SiHa cells were either transfected with si16E6/E7 or siCtrl1 and grown for 72 h or were treated for 24 h with 7.5 mM Metformin or DMSO, each in biological triplicates. Sample preparation and MS analyses were performed as previously described [49]. The MS‐based proteomics data for cells transfected with si16E6/E7 or siCtrl1 have been deposited to the ProteomeXchange Consortium via the PRIDE [50] partner repository with the data set identifier PXD061861 and raw mass spectra files were analyzed as described before [39]. Similarly, the MS‐based proteomics data for cells treated with Metformin or DMSO are deposited to the ProteomeXchange Consortium with the data set identifier PXD011095 and raw mass spectra files were analyzed as detailed elsewhere [49].

Gene set enrichment analysis (GSEA) was performed using GSEA v4.3.3 [51, 52] and the gene sets from the C2 collection (curated gene sets) were obtained from the Molecular Signature Database (MSigDB, c2.all.v2024.1.Hs.symbols.gmt) [53, 54]. Preranked enrichment analysis was conducted using the signed log_10_ *p* values of 4649 detected proteins (siE6/E7 versus siCtrl1, Table S2) or 5929 detected proteins (Metformin versus DMSO, Table S3) [33] based on *n* = 3 samples per condition with the following parameters: number of permutations, 1000; enrichment statistic, weighted; gene set size filters, min = 15, max = 600; normalized mode, meandiv; seed for permutation, timestamp. Signed log_10_ *p* values were calculated as follows: signed log_10_ *p* value = abs(log_10_(*p* value) × 10) × sign(log_2_FC).

### 2.10 Statistical Analyses

GraphPad Prism 10.2.3 (GraphPad Software Inc.) was used to determine statistical significance by two‐sided unpaired *t*‐tests or one‐way/two‐way analysis of variance (ANOVA), followed by Tukey's test for multiple comparisons, as appropriate. Statistical significance is indicated as *p ≤ 0.05, **p ≤ 0.01, and ***p ≤ 0.001.

## 3 Results

### 3.1 E6/E7 Repression by Metformin or by RNAi Differentially Affects Cell Cycle Regulators in HPV‐Positive Cancer Cells

**Figure 1:**
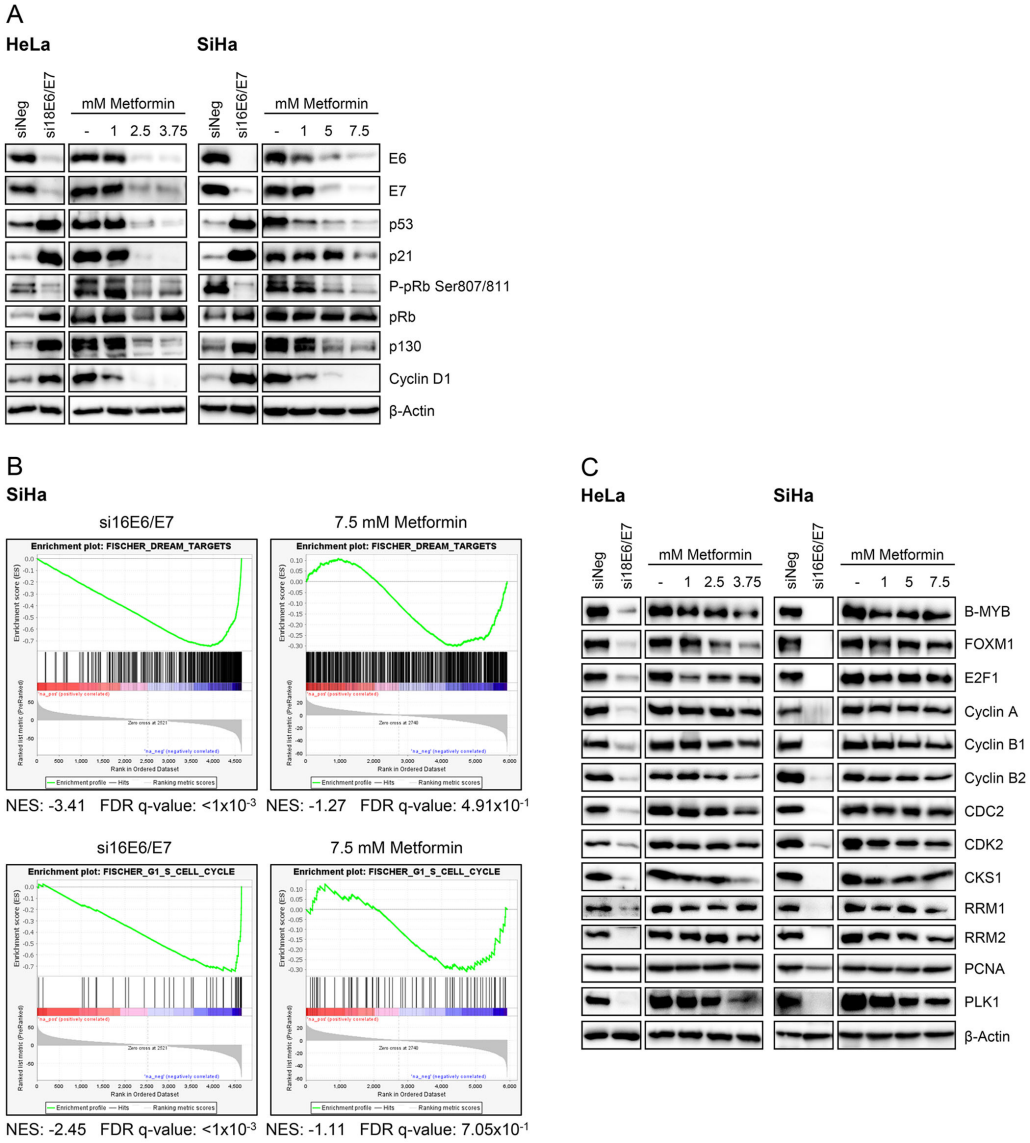
Repression of E6/E7 by Metformin treatment or RNAi differentially affects expression of cell cycle regulators in HPV‐positive cancer cells. (A) HeLa or SiHa cells were either transfected with si18E6/E7 or si16E6/E7, or control siRNA (siNeg) and grown for 72 h, or treated with the indicated concentrations of Metformin for 24 h and examined by immunoblot for E6, E7, p53, p21, P‐pRb Ser807/811, pRb, p130, Cyclin D1, and β‐Actin protein levels. (B) SiHa cells were either transfected with si16E6/E7 or control siRNA (siCtrl1) and grown for 72 h, or treated with 7.5 mM Metformin or DMSO for 24 h. Signed log_10_ *p* values from proteome analyses comparing siE6/E7 versus siCtrl1 or Metformin versus DMSO were used as input for preranked gene set enrichment analysis (GSEA) with 1000 permutations using the C2 collection. Enrichment plots for the gene sets FISCHER_DREAM_TARGETS [55] and FISCHER_G1_S_CELL_CYCLE [55] are shown. Proteins uniquely detected in either the Metformin or the siE6/E7 data set exhibited a distribution largely similar to those detected in both data sets (not shown). NES, normalized enrichment score; FDR, false discovery rate. (C) HeLa or SiHa cells were treated as described for subfigure (A). Immunoblot analyses of B‐MYB, FOXM1, E2F1, Cyclin A, Cyclin B1, Cyclin B2, CDC2, CDK2, CKS1, RRM1, RRM2, PCNA, PLK1, and β‐Actin protein levels are shown.

To gain insights into the differential phenotypic responses of HPV‐positive cancer cells to E6/E7 repression by RNAi (inducing growth inhibition and senescence) or by Metformin treatment (inducing growth inhibition, but no senescence) [33], the expression of key factors involved in cell cycle regulation and senescence control was comparatively analyzed. In line with previous findings [37], RNAi‐induced E6/E7 repression led to a pronounced increase in p53, p21, p130, and pRb levels, and a concomitant downregulation of phosphorylated pRb in HPV18‐positive HeLa and HPV16‐positive SiHa cells (Figure 1A). Moreover, the levels of Cyclin D1, which are upregulated in senescent cells [56], were increased upon E6/E7 silencing. Importantly, and in strong contrast to RNAi‐mediated E6/E7 repression, the dose‐dependent downregulation of E6/E7 by Metformin treatment was not accompanied by an increase in the levels of these factors but rather led to a dose‐dependent downregulation of p53, p21, p130, and Cyclin D1.

The observation that Metformin inhibits E6/E7 expression but does not lead to the anticipated and well‐documented increase in p53 or p21 levels [28, 57] implies that Metformin may affect alternative pathways that can override the p53 stabilization in response to E6 inhibition. We found that *TP53* mRNA levels remained unchanged upon Metformin treatment in both HeLa and SiHa cells (Figure S1A), indicating a post‐translational mechanism of p53 repression. Furthermore, Metformin also counteracted p53 and p21 accumulation triggered by RNAi‐mediated knockdown of E6AP (the E6‐associated ubiquitin ligase responsible for targeting p53 for proteasomal degradation) [58] or E6/E7 in SiHa cells (Figure S1B), indicating that it can override the typical downstream effects of E6 or E6AP inhibition on p53. Collectively, these data suggest that Metformin affects p53 expression through E6‐ and E6AP‐independent pathways. To corroborate this further, we analyzed HPV‐negative cells, in which, contrary to HPV‐positive cells, p53 degradation is primarily mediated by the cellular E3 ligase Mdm2 [59]. We found that Metformin treatment reduced basal p53 and p21 protein levels in a dose‐dependent manner in U2OS osteosarcoma cells (Figure S1C). Moreover, Metformin efficiently counteracted p53 and p21 induction by the Mdm2 inhibitor Nutlin‐3 [60] in both U2OS and HCT116 colon cancer cells (Figure S1D), further supporting the notion that Metformin can downregulate p53 via an HPV‐independent mechanism.

Given the differential effects of E6/E7 knockdown or Metformin treatment, we next used MS‐based proteomics to analyze global proteomic changes in SiHa cells after siRNA‐mediated E6/E7 silencing (Table S2) or Metformin treatment (Table S3) [33]. GSEA [51, 52] using the C2 database [53, 54] was employed to identify significantly enriched pathways associated with these perturbations. siRNA‐mediated knockdown of E6/E7 led to a strong negative enrichment of the FISCHER_DREAM_TARGETS [55] gene set, indicative for an activation of the p53‐p21‐DREAM (dimerization partner, RB‐like, E2F, and multi‐vulval class B complex; containing p130) pathway and an increase in DREAM complex activity, as the complex functions as transcriptional repressor (Figure 1B, upper left panel). Further, E6/E7 knockdown led to a strong negative enrichment of the FISCHER_G1_S_CELL_CYCLE [55] gene set, which is upregulated during cell cycle progression at the G1/S transition (Figure 1B, lower left panel). This is consistent with the notion that the p53‐p21‐DREAM and p53‐p21‐pRb signaling pathways can support senescence induction by repressing transcription of genes critical for cell cycle progression, thereby leading to cell cycle arrest [61, 62, 63]. In contrast, Metformin‐treated cells exhibited considerably weaker, non‐significant negative enrichment of these gene sets (Figure 1B, right panels). To further support these findings using an independent experimental approach, the expression of several DREAM and/or pRb targets, including proteins detected in the proteomics screen (Table S4) as well as additional targets, was analyzed by immunoblot analyses (Figure 1C). Consistent with the results obtained from proteome analyses, well‐known DREAM and/or pRb targets such as B‐MYB, FOXM1, E2F1, Cyclin A, Cyclin B1, Cyclin B2, CDC2 (CDK1), CDK2, CKS1, RRM1, RRM2, PCNA, and PLK1 [61, 64, 65] were downregulated upon siRNA‐mediated E6/E7 silencing, but were not or only slightly affected by Metformin treatment in both HeLa and SiHa cells.

Collectively, these findings indicate that E6/E7 silencing by RNAi leads to a pronounced downregulation of proteins promoting cell cycle progression, which occurs, if at all, to a much lesser extent upon Metformin treatment, albeit E6/E7 expression is effectively blocked under both growth‐inhibitory conditions.

### 3.2 Metformin Induces E6/E7‐Independent Cell Cycle Arrest in HPV‐Positive Cancer Cells

**Figure 2:**
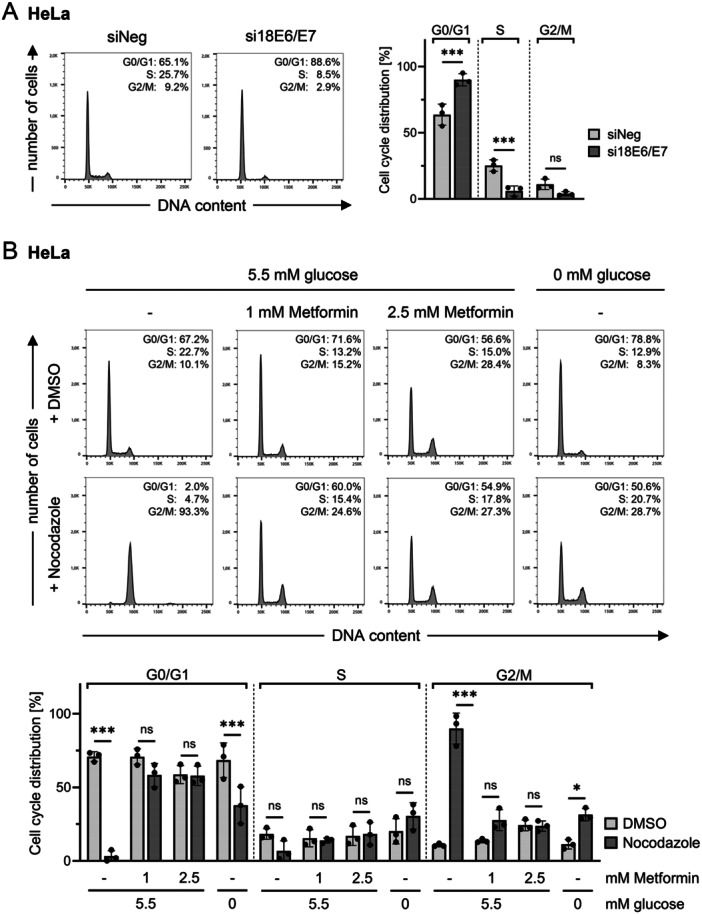
Metformin blocks proliferation of HeLa cells without pronounced accumulation of cells in a specific cell cycle phase. (A) HeLa cells were transfected with si18E6/E7 or control siRNA (siNeg), and grown for 72 h. Cell cycle profiles were analyzed by flow cytometry. Left panel: Results of one representative experiment with corresponding quantifications of the cell populations across the individual cell cycle phases. Right panel: Cell cycle distribution measured in three independent experiments, shown as mean percentages ± SD. Statistically significant differences were calculated using two‐way ANOVA with Tukey's test for multiple comparisons. Comparisons between siNeg and si18E6/E7 within each cell cycle phase are indicated. ns, not significant, ***p ≤ 0.001. (B) HeLa cells were treated with the indicated concentrations of Metformin or cultivated in medium containing no glucose (0 mM) for 48 h. Nocodazole at a final concentration of 0.1 µg/mL or DMSO (solvent control) was added for the last 24 h of treatment. Cell cycle profiles were analyzed by flow cytometry. Upper panel: Results of one representative experiment with corresponding quantifications of the cell populations across the individual cell cycle phases. Lower panel: Cell cycle distribution measured in three independent experiments, shown as mean percentages ± SD. Statistically significant differences were calculated using two‐way ANOVA with Tukey's test for multiple comparisons. Comparisons between DMSO and Nocodazole for the same cell cycle phase within each condition are indicated. ns, not significant, *p ≤ 0.05, ***p ≤ 0.001.

In view of the differential regulation of cell cycle‐promoting factors upon E6/E7 repression by RNAi or by Metformin, comparative cell cycle analyses were performed. Consistent with previous findings [37, 66], RNAi‐mediated E6/E7 silencing induced an efficient G1 arrest in HeLa (Figure 2A) and SiHa cells (Figure S2A). In strong contrast, Metformin treatment did not induce a pronounced G1 arrest and led to a moderate increase in G2/M populations (Figures 2B and S2B), despite effective E6/E7 repression upon higher doses (Figure 1A). Although the cell cycle profiles of HeLa and SiHa cells did not reveal a pronounced arrest in a specific phase of the cell cycle, cells were effectively growth inhibited by Metformin treatment as shown in proliferation assays (Figure S3). This notion was further supported by co‐treatment experiments with Nocodazole, a microtubule polymerization inhibitor that blocks proliferating cells in the G2/M phase [67]. Whereas proliferating control cells showed an efficient G2/M arrest upon Nocodazole treatment, cells co‐treated with Metformin and Nocodazole did not or only weakly increase G2/M populations (Figures 2B and S2B). Importantly, low doses of Metformin (1 mM), which were ineffective in E6/E7 repression (Figure 1A), still induced a cell cycle arrest and inhibited the proliferation of both HeLa (Figure 2B) and SiHa cells (Figures S2B and S3). This latter finding shows that Metformin can exert its anti‐proliferative effects despite the presence of E6 and E7.

Glucose deprivation has been shown to act anti‐proliferative in many cancer cell lines [68, 69, 70, 71] and, similarly to 1 mM Metformin, only marginally affects E6/E7 expression in HPV‐positive cancer cells, if at all (Figure S4) [36]. Therefore, the effects of Metformin on the cell cycle of HPV‐positive cancer cells were compared to those of glucose deprivation. Glucose withdrawal from the cell culture medium resulted in a moderate increase of G1 populations in both HeLa and SiHa cells (compared to proliferating control cells cultivated under 5.5 mM glucose) and acted strongly anti‐proliferative. This was indicated by inefficient induction of G2/M arrest upon Nocodazole treatment in cell cycle analyses (Figures 2B and S2B) and a strong growth inhibition in proliferation assays (Figure S3).

Overall, these findings show that Metformin effectively inhibits the proliferation of HPV‐positive cancer cells without inducing a pronounced arrest in a specific cell cycle phase. This effect appears to be independent of E6/E7 repression and is also observed upon glucose deprivation.

### 3.3 Metformin‐Induced Growth Arrest Protects Cancer Cells From Etoposide‐Induced Senescence

**Figure 3:**
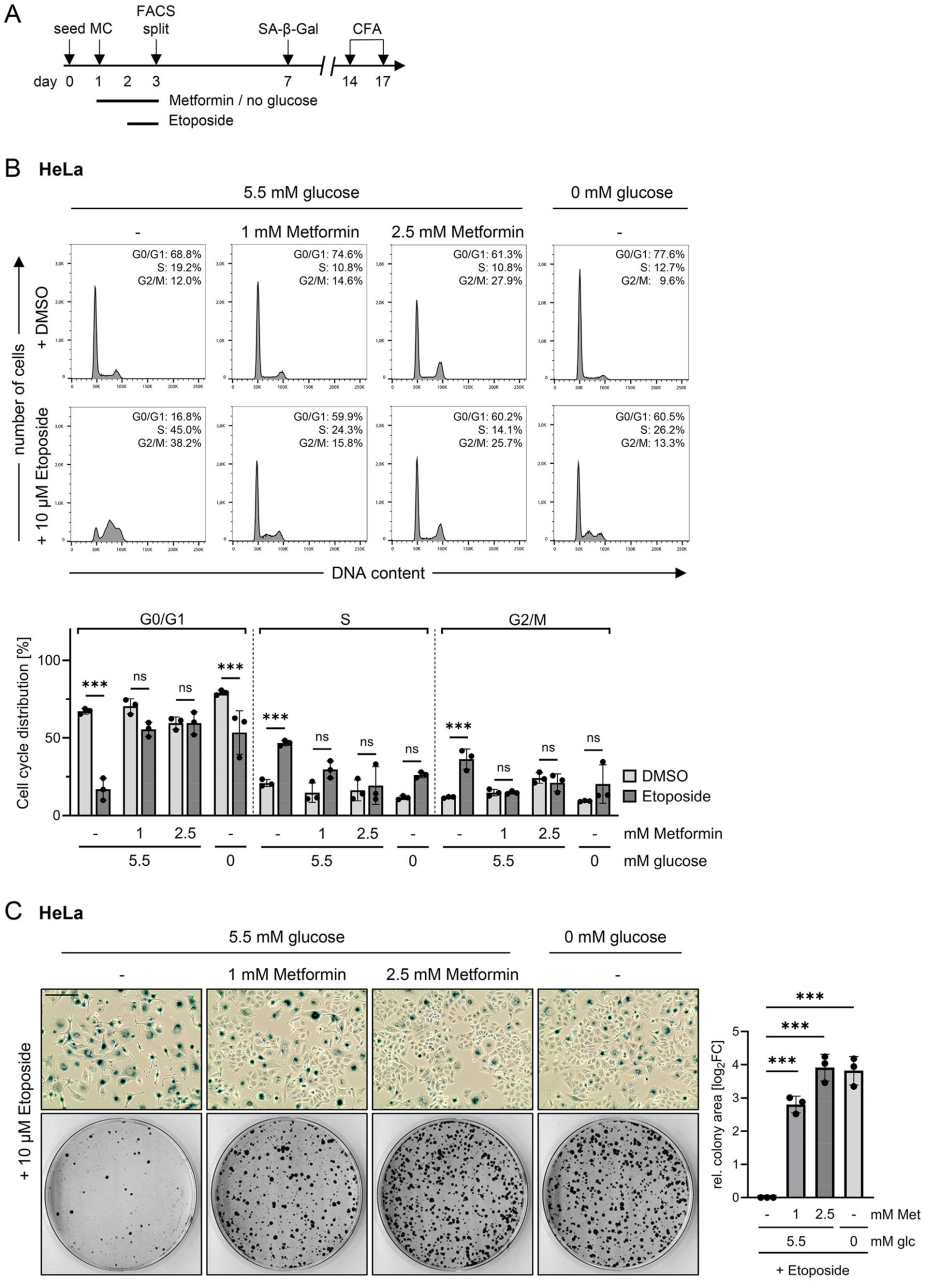
Metformin treatment or glucose deprivation protects HeLa cells from the pro‐senescent effects of Etoposide. (A) Treatment scheme: Cells were treated with the indicated concentrations of Metformin or cultivated in medium containing no glucose (0 mM) for 48 h. Etoposide or DMSO (solvent control) was added for the last 24 h of treatment. Subsequently, cells were either harvested for flow cytometry analyses (FACS), or split and further cultivated in drug‐free medium for senescence assays (SA‐β‐Gal staining) or colony formation assays (CFAs) after the indicated time periods. MC, medium change. (B) Cell cycle profiles of HeLa cells were analyzed by flow cytometry. Upper panel: Results of one representative experiment with corresponding quantifications of the cell populations across the individual cell cycle phases. Lower panel: Cell cycle distribution measured in three independent experiments, shown as mean percentages ± SD. Statistically significant differences were calculated using two‐way ANOVA with Tukey's test for multiple comparisons. Comparisons between DMSO and Etoposide for the same cell cycle phase within each condition are indicated. ns, not significant, ***p ≤ 0.001. (C) Left panel: Corresponding senescence assays (upper panels; SA‐β‐Gal staining, blue; scale bar: 200 μm) and CFAs (lower panels). Right panel: Quantification of colony areas from three independent CFA experiments. Log_2_‐transformed fold changes (log_2_FC) of mean colony area ± SD are shown. Statistically significant differences were calculated using one‐way ANOVA with Tukey's test for multiple comparisons. Comparisons between Etoposide‐treated cells (log_2_FC = 0) and each other condition are indicated. ***p ≤ 0.001. glc, glucose; Met, Metformin; rel, relative.

Etoposide can act as a pro‐senescent chemotherapeutic agent and, in addition, has been evaluated in clinical studies for its safety and efficacy as an adjunct therapy in cervical cancer [72, 73]. We previously observed that Metformin has the potential to block senescence induction by Etoposide in HPV‐positive cancer cells [33]. Etoposide acts as an inhibitor of nuclear topoisomerase II which ultimately leads to senescence of proliferating cells by inducing DNA damage in the S phase of the cell cycle [74, 75]. We hypothesized that the ability of Metformin to induce a pronounced proliferation arrest (Figures 2B and S2B) may underlie the protective effects towards Etoposide‐induced senescence. Therefore, the combination of both agents was investigated following the treatment scheme depicted in Figure 3A. Cell cycle analyses revealed that, as expected [74, 76, 77, 78], Etoposide monotreatment induced an accumulation of cells in S phases and a G2/M arrest, in both HeLa (Figure 3B) and SiHa cells (Figure S5A). Interestingly, these Etoposide‐induced effects were no longer observable upon pre‐treatment with Metformin and cell cycle profiles remained mostly unchanged. Similarly, pre‐cultivation of these cells in glucose‐free medium was sufficient to largely impair Etoposide‐induced cell cycle arrest (Figures 3B and S5A).

Further, both Metformin treatment and pre‐cultivation in glucose‐free medium efficiently protected HPV‐positive cancer cells from Etoposide‐induced senescence. Specifically, HeLa and SiHa cells treated with Etoposide in the presence of glucose stained positive for the senescence marker Senescence‐Associated‐β‐Galactosidase (SA‐β‐Gal) and exhibited the typical morphological characteristics of senescent cells (cytoplasmic extensions, cellular enlargement and flattening) [79, 80]. In contrast, either in the presence of Metformin or in the absence of glucose, a substantial portion of cells stained SA‐β‐Gal‐negative and lacked the morphological signs of senescence (Figures 3C and S5B, upper panels for each cell line). Since senescence is classically defined as an irreversible growth arrest [30], the proliferative potential of the cells after Etoposide and Metformin removal from the cell culture medium was analyzed in CFAs. In line with their increased ability to evade Etoposide‐induced senescence, both Metformin treatment and glucose deprivation allowed a substantially higher outgrowth of cells after drug removal (Figures 3C and S5B, lower panels for each cell line) [33].

The finding that the growth‐inhibitory activity of lower doses of Metformin (1 mM) in HPV‐positive cancer cells is not linked to viral E6/E7 repression (Figures 1A and S3) raised the possibility that the observed effects are not restricted to HPV‐positive cancer cells. Indeed, corresponding results were obtained in HPV‐negative MCF‐7 breast cancer and U2OS osteosarcoma cells, where pre‐treatment with Metformin also prevented Etoposide‐induced senescence (Figure S6, upper panels for each cell line) and resulted in increased colony formation upon Etoposide and Metformin removal (Figure S6, lower panels for each cell line).

Taken together, these results indicate that pre‐treatment with Metformin, similar to pre‐cultivation in glucose‐free medium, allows HPV‐positive cancer cells to escape Etoposide‐induced senescence. This protective effect of Metformin is linked to its pronounced growth inhibitory activity, which prevents Etoposide‐induced DNA damage during the S phase passage. Furthermore, this cellular response does not depend on Metformin‐induced viral E6/E7 repression in HPV‐positive cancer cells and is conserved in HPV‐negative cancer cells of different histopathological backgrounds.

### 3.4 Metformin Can Have Both Pro‐ and Anti‐Apoptotic Effects When Combined With Cisplatin in HPV‐Positive Cancer Cells

As demonstrated, the combination of Metformin with Etoposide may have unfavorable effects in the context of cancer therapy, as it can prevent senescence induction. Therefore, we next aimed to investigate whether Metformin also affects the efficacy of Cisplatin, which can act either pro‐senescent or pro‐apoptotic [10, 39, 81] and plays a key role in the treatment of advanced and recurrent cervical cancer [5].

**Figure 4:**
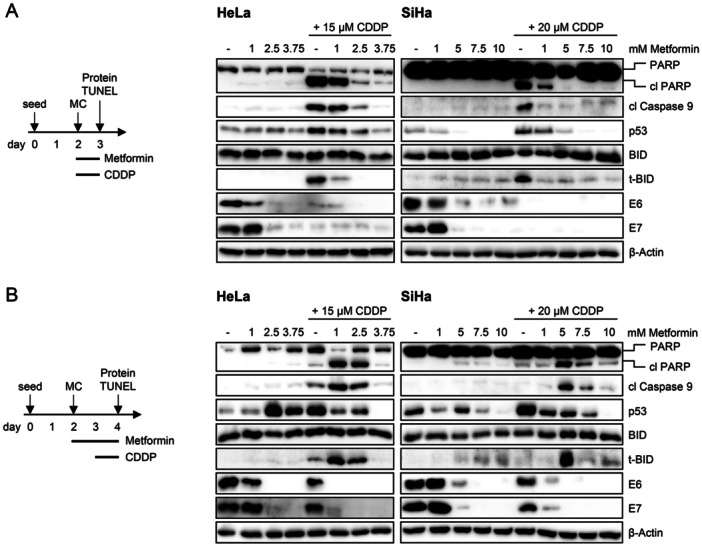
Metformin can diminish or enhance the pro‐apoptotic effects of Cisplatin in HPV‐positive cancer cells, depending on treatment conditions. (A) Left panel: Treatment scheme; HeLa or SiHa cells were treated with the indicated concentrations of Metformin and/or Cisplatin (CDDP) for 24 h. Subsequently, cells were harvested for protein analyses. MC, medium change. Right panel: Corresponding immunoblot analyses of PARP, cleaved (cl) PARP, cl Caspase 9, p53, BID, t‐BID, E6, E7, and β‐Actin protein levels. (B) Left panel: Treatment scheme; HeLa or SiHa cells were treated with the indicated concentrations of Metformin for 48 h. CDDP was added for the last 24 h of treatment, if indicated. Subsequently, cells were harvested for protein analyses. Right panel: Corresponding immunoblot analyses of PARP, cl PARP, cl Caspase 9, p53, BID, t‐BID, E6, E7, and β‐Actin protein levels.

Cisplatin monotreatment for 24 h acted pro‐apoptotic in HeLa and SiHa cells, as indicated by strongly increased levels of the apoptosis markers cleaved poly(ADP‐ribose) polymerase (PARP) and cleaved Caspase 9 (Figure 4A), as well as a high number of TUNEL‐positive cells (Figure S7). Cisplatin‐induced apoptosis was linked to an increase in p53 levels as well as cleavage of the BH3‐interacting domain death agonist (BID) protein leading to truncated BID (t‐BID), a key effector of Cisplatin‐mediated apoptosis in HPV‐positive cancer cells [39]. Interestingly, however, simultaneous treatment with Cisplatin and Metformin for 24 h resulted in pronounced protective effects against Cisplatin‐induced apoptosis. This was evidenced by a dose‐dependent downregulation of cleaved PARP, cleaved Caspase 9, and t‐BID, as well as p53 (Figure 4A), and by a strong decrease in TUNEL‐positive cells (Figure S7), compared to Cisplatin treatment alone. These findings show that Metformin can efficiently counteract Cisplatin‐induced apoptosis in HPV‐positive cancer cells, if both drugs are applied simultaneously.

In contrast, pre‐treatment with Metformin for 24 h before adding Cisplatin for another 24 h (Figure 4B) resulted in partly opposing effects. Whereas Cisplatin monotreatment was sublethal under these conditions, potentially due to less optimal cellular growth conditions, lower doses of Metformin (1 and 2.5 mM [HeLa] or 5 mM [SiHa]) significantly enhanced apoptosis in both HeLa and SiHa cells when combined with Cisplatin. This was evidenced by a pronounced upregulation of cleaved PARP, cleaved Caspase 9, and t‐BID (Figure 4B) as well as an increased number of TUNEL‐positive cells (Figure S8A). Remarkably, these cooperative pro‐apoptotic effects were diminished at higher Metformin concentrations (3.75 mM [HeLa] or 7.5 and 10 mM [SiHa]), as indicated by lower levels of cleaved PARP, cleaved Caspase 9, and t‐BID (Figure 4B), and decreasing numbers of apoptotic cells observed in light microscopy images taken at this time point (Figure S8B). Furthermore, whereas Cisplatin monotreatment led to p53 upregulation also under these experimental conditions, the combination with Metformin counteracted the Cisplatin‐induced upregulation of p53 in a dose‐dependent manner (Figure 4B).

Of note, we observed fluctuating p53 levels in control cells treated with Metformin alone. Despite the potential of Metformin to downregulate p53 (Figures 1A and S1) and to counteract Cisplatin‐induced p53 upregulation (Figure 4), treatment with Metformin alone resulted in increased total p53 levels at certain concentrations (Figure 4B). To investigate this further, the effects of Metformin on p53 were analyzed in dose titration experiments over time (Figure S9). Interestingly, we observed an initial downregulation of p53 at 24 h, whereas longer Metformin treatment resulted in an increase in total p53 levels as well as post‐translationally modified p53 forms (phospho‐p53 Ser15 and acetyl‐p53 Lys382) after 48 h (HeLa) or 72 h (SiHa), particularly at 2.5 mM (HeLa) and 5 mM Metformin (SiHa). At further elevated Metformin doses (3.75 mM [HeLa] or 7.5 mM [SiHa]), the levels of p53 and its modified forms decreased again (Figure S9). These findings indicate that Metformin can decrease or increase p53 levels in a time‐ and dose‐dependent manner, which could explain the fluctuations of p53 levels that were observed for Metformin monotreatment under certain experimental conditions (Figure 4).

Additionally, we investigated whether glucose deprivation, which had the same protective effects as Metformin treatment against Etoposide‐induced senescence (Figures 3 and S5), also mimics the effects of Metformin when combined with Cisplatin. We found that glucose deprivation neither protected HeLa cells from Cisplatin‐induced apoptosis when applied simultaneously with Cisplatin (Figure S10A) nor enhanced Cisplatin‐induced apoptosis when cells were glucose‐starved before treatment (Figure S10B).

Collectively, these data show that Metformin exerts complex effects on HPV‐positive cancer cells and, interestingly, can either inhibit or promote the pro‐apoptotic activity of Cisplatin, depending on treatment conditions.

### 3.5 p53 Plays a Key Role in the Interplay Between Metformin and Cisplatin in HPV‐Positive Cancer Cells

p53 has been reported to be critically involved in Cisplatin‐induced apoptosis [82, 83]. Therefore, we investigated the possible involvement of p53 in the agonistic or antagonistic effects of Metformin on Cisplatin‐mediated apoptosis in HPV‐positive cancer cells.

**Figure 5:**
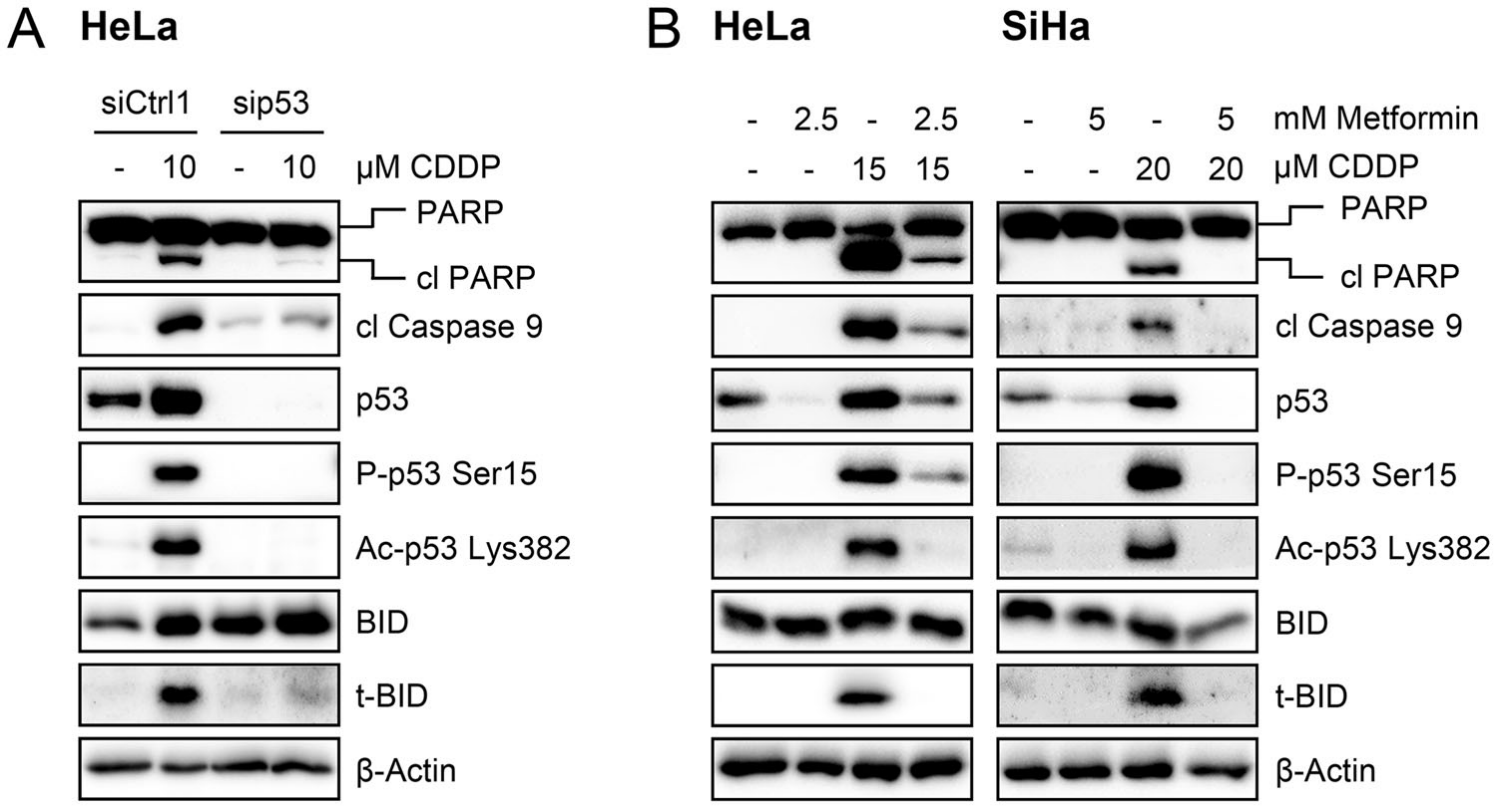
Metformin efficiently counteracts Cisplatin‐mediated upregulation of p53. (A) HeLa cells were transfected with sip53 or control siRNA (siCtrl1). Forty‐eight hours after transfection, the medium was changed and cells were treated with Cisplatin (CDDP) for 24 h. Protein levels of PARP, cleaved (cl) PARP, cl Caspase 9, p53, P‐p53 Ser15, Ac‐p53 Lys382, BID, t‐BID, and β‐Actin were measured by immunoblot analyses. (B) HeLa or SiHa cells were treated with the indicated Metformin and/or Cisplatin concentrations for 24 h, following the treatment scheme depicted in Figure 4A. Protein levels of PARP, cl PARP, cl Caspase 9, p53, P‐p53 Ser15, Ac‐p53 Lys382, BID, t‐BID, and β‐Actin were measured by immunoblot analyses.

The pro‐apoptotic effects of Cisplatin, as mirrored by the upregulation of cleaved PARP, cleaved Caspase 9, and t‐BID, were linked to a strong increase in the levels of p53 and its post‐translationally modified forms known to be associated with DNA damage [84, 85, 86, 87], phospho‐p53 Ser15 and acetyl‐p53 Lys382, in both HeLa (Figure 5A) and SiHa cells (Figure S11). Functional analyses revealed that the upregulation of the apoptosis markers by Cisplatin is p53‐dependent, as it was efficiently blocked by RNAi‐mediated p53 downregulation (Figures 5A and S11). Importantly, under conditions where Metformin inhibited Cisplatin‐induced apoptosis (Figure 4A), concomitant Metformin treatment efficiently counteracted the Cisplatin‐induced upregulation of p53 and its post‐translationally modified forms. Moreover, this effect strictly correlated with a decrease of cleaved PARP, cleaved Caspase 9, and t‐BID levels, in both HeLa and SiHa cells (Figure 5B). These observations indicate that the antagonistic effect of Metformin on Cisplatin‐induced apoptosis could depend on its ability to counteract p53 induction. In further support of this notion, the Metformin‐mediated protection against Cisplatin‐induced apoptosis was consistently associated with strongly impaired p53 induction under different experimental conditions (Figure 4).

**Figure 6:**
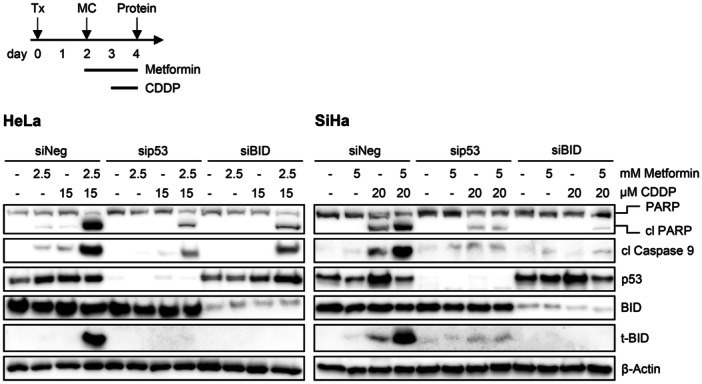
p53 and BID/t‐BID are critical for the enhancing effects of Metformin on Cisplatin‐induced apoptosis in HPV‐positive cancer cells. Upper panel: Treatment scheme; HeLa or SiHa cells were transfected with sip53, siBID, or control siRNA (siNeg). Forty‐eight hours after transfection, cells were treated with the indicated concentrations of Metformin for 48 h. Cisplatin (CDDP) was added for the last 24 h of treatment, if indicated. Subsequently, cells were harvested for protein analyses. Tx, transfection; MC, medium change. Lower panel: Corresponding immunoblot analyses of PARP, cleaved (cl) PARP, cl Caspase 9, p53, BID, t‐BID, and β‐Actin protein levels.

Next, we investigated the role of p53 in the agonistic effects of Metformin pre‐treatment on Cisplatin‐induced apoptosis. Under these conditions, the combined treatment with Metformin and Cisplatin led to strongly increased expression levels of cleaved PARP, cleaved Caspase 9, and t‐BID, in both HeLa and SiHa cells (Figure 4B). Interestingly, this response was efficiently blocked when p53 was concomitantly downregulated by RNAi (Figures 6 and S12). Of note, although the latter finding indicates that the increased pro‐apoptotic effects of combining Metformin and Cisplatin are clearly p53‐dependent, the increase in apoptosis markers did not strictly correlate with total p53 levels (Figures 4D, 6, and S12). Additionally, RNAi‐mediated BID/t‐BID downregulation led to similar results as p53 repression (Figure 6), indicating that the enhancing effects of Metformin on Cisplatin‐induced apoptosis are both p53‐ and BID/t‐BID‐dependent.

Taken together, these data show that the induction of p53 is crucial for the pro‐apoptotic effects of Cisplatin in HPV‐positive cancer cells. Furthermore, in cells pre‐treated with Metformin, Metformin can increase the pro‐apoptotic activity of Cisplatin in a p53‐and BID/t‐BID‐dependent manner. On the other hand, when Cisplatin and Metformin are applied simultaneously, Metformin can inhibit Cisplatin‐induced apoptosis. This latter effect is linked to a severe impairment of the Cisplatin‐induced p53 increase by Metformin and occurs in a dose‐ and time‐dependent manner.

### 3.6 Metformin and Cisplatin Can Cooperate to Inhibit Long‐Term Growth of HPV‐Positive Cancer Cells

It is important to note that the discrepant results on the apoptosis response upon combining Metformin and Cisplatin were obtained upon harvesting cells immediately after treatment. Therefore, we next assessed the long‐term consequences of the experimental conditions under which Metformin can inhibit (Figure 4A) or promote (Figure 4B) the pro‐apoptotic activity of Cisplatin. In view of the potential of Cisplatin to act both pro‐apoptotic and pro‐senescent [81], HeLa and SiHa cells were treated with increasing doses of Cisplatin and/or Metformin and subsequently analyzed by senescence assays and CFAs.

**Figure 7:**
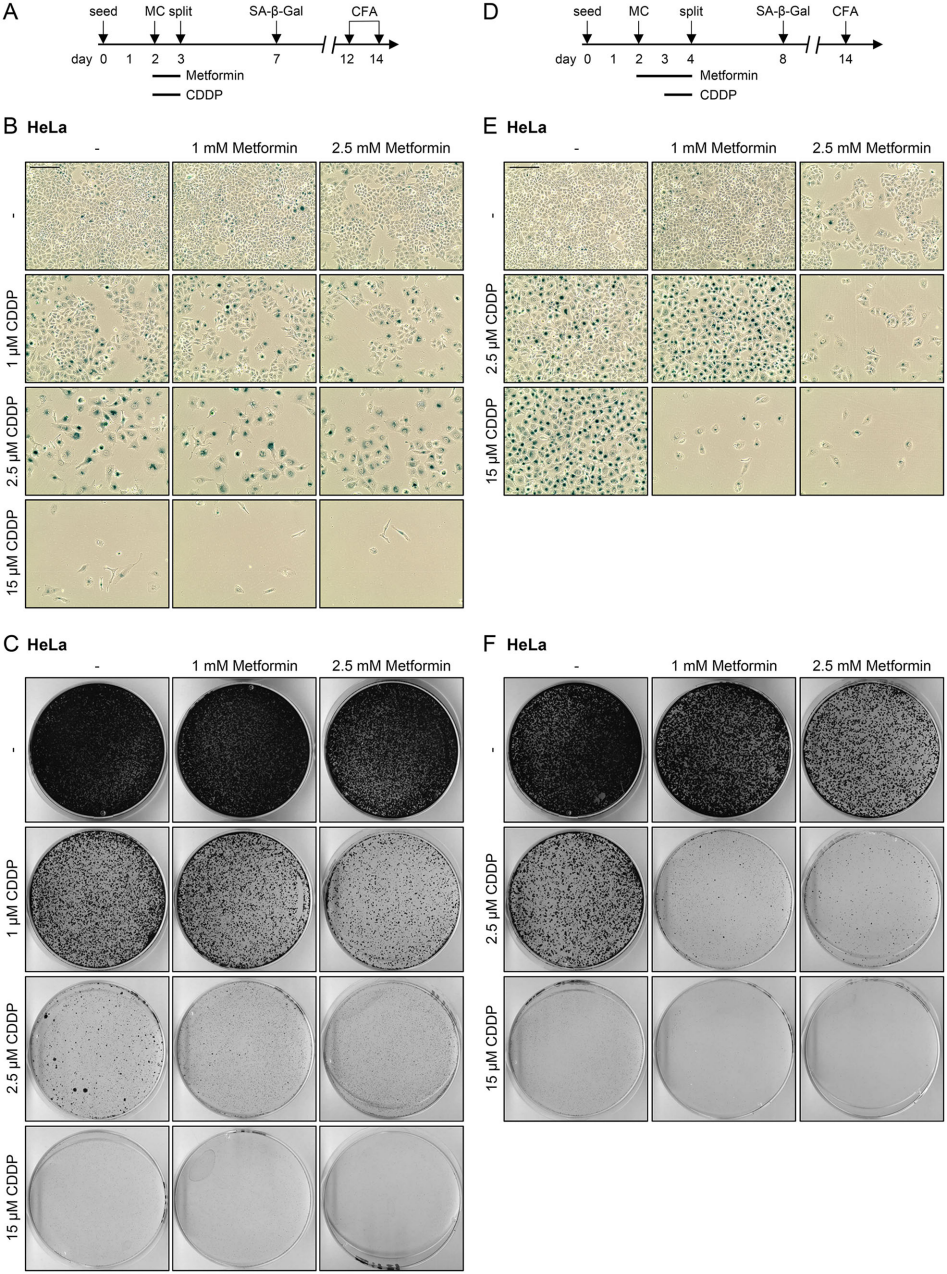
Metformin and Cisplatin can cooperate to inhibit long‐term growth of HeLa cells. (A) Treatment scheme: Cells were treated with the indicated concentrations of Metformin and/or Cisplatin (CDDP) for 24 h. Subsequently, cells were split and further cultivated in drug‐free medium for senescence assays (SA‐β‐Gal staining) or colony formation assays (CFAs) after the indicated time periods. MC, medium change. Corresponding (B) senescence assays (SA‐β‐Gal staining, blue; scale bar: 200 μm) and (C) CFAs of HeLa cells treated as depicted in subfigure (A). (D) Treatment scheme: Cells were treated with the indicated concentrations of Metformin for 48 h. CDDP was added for the last 24 h of treatment, if indicated. Subsequently, cells were split and further cultivated in drug‐free medium for senescence assays (SA‐β‐Gal staining) or CFAs after the indicated time periods. Corresponding (E) senescence assays (SA‐β‐Gal staining, blue; scale bar: 200 μm) and (F) CFAs of HeLa cells treated as depicted in subfigure (D).

When cells were treated following the scheme depicted in Figure 7A, Cisplatin alone induced pro‐senescent effects at low doses, in both HeLa (Figure 7B) and SiHa cells (Figure S13A). With increasing doses, Cisplatin tightened the senescence response by progressively diminishing the numbers of surviving, non‐senescent cells and inhibiting cellular outgrowth in CFAs (Figures 7B,C, S13A,B, and S14A,B). Further, at the highest tested concentration (15 μM [HeLa] or 20 μM [SiHa]), Cisplatin acted strongly cytotoxic. Interestingly, and in remarkable contrast to the strong protection against Cisplatin‐induced apoptosis observed immediately after treatment (Figures 4A and S7), simultaneous treatment with Cisplatin and Metformin did not result in protection of HeLa and SiHa cells at later time points, but rather resulted in an additional reduction of their colony formation capacities (Figures 7B,C, S13A,B, and S14A,B). These latter findings indicate that the protective effect of Metformin against Cisplatin‐induced apoptosis, which is observed immediately after treatment (Figure 4A), is only transient. In further support of this conclusion, the repression of Cisplatin‐induced apoptosis by simultaneous Metformin treatment was no longer observed 24 h after drug release from the cell culture medium in both HeLa and SiHa cells (Figure S15). Rather, apoptosis induction, as evidenced by the upregulation of cleaved PARP and cleaved Caspase 9 as well as strongly elevated p53 levels, was comparable to Cisplatin monotreatment.

When cells were treated following the experimental scheme depicted in Figure 7D, Cisplatin alone primarily led to increasing pro‐senescent effects with increasing doses in both HeLa and SiHa cells. This was reflected in a progressive decrease in the number of non‐senescent cells (Figures 7E and S13C) and a corresponding decrease in cellular outgrowth in CFAs (Figures 7F, S13D, and S14C,D). Importantly, we observed strong cooperative effects between Cisplatin and Metformin in this treatment condition. At a dose of 2.5 μM Cisplatin, co‐treatment with 1 mM Metformin substantially tightened the senescence response, as non‐senescent cells were virtually undetectable. Furthermore, 2.5 mM (HeLa) or 5 mM Metformin (SiHa) combined with Cisplatin resulted in a reduction in overall cell numbers, indicating efficient induction of cell death in both cell lines (Figures 7E and S13C). Interestingly, at higher doses of Cisplatin (15 µM [HeLa] or 20 µM [SiHa]), co‐treatment with lower Metformin concentrations (1 mM) was sufficient to switch the phenotype of treated cells from senescence to cell death. These anti‐proliferative effects were confirmed in CFAs, in which the combination treatments significantly suppressed cellular outgrowth (Figures 7F, S13D, and S14C,D).

Overall, these results indicate that the protective effect of Metformin against Cisplatin‐induced apoptosis, which is seen in certain treatment conditions immediately after treatment, is transient. Moreover, we show that, especially upon Metformin pre‐treatment, Cisplatin and Metformin can strongly cooperate to inhibit the proliferation of HPV‐positive cancer cells over time. This effect is linked to an enhanced senescence or cell death response and also a phenotypic switch from senescence to cell death, which is not observed in cells that were not pre‐treated with Metformin.

## 4 Discussion

Novel strategies to improve treatment options for HPV‐positive cancers are urgently required. Metformin is being considered for repurposing as an anti‐cancer agent in the therapy of various cancer entities [17, 24, 88], including HPV‐positive cancers [18, 20, 89], but this is under controversial debate. It is well established that the proliferation of HPV‐positive cancer cells is dependent on the continuous expression of the HPV *E6/E7* oncogenes [26]. In this light, it is interesting that Metformin can strongly downregulate viral E6/E7 expression and block the growth of HPV‐positive cancer cells [33]. This raises the possibility that Metformin may act anti‐proliferative in HPV‐positive cancer cells by interfering with the expression of the growth‐promoting viral *E6/E7* oncogenes.

However, the results of our study argue against this notion as we found that the mechanisms underlying the growth inhibition of HPV‐positive cancer cells induced by either RNAi‐mediated E6/E7 silencing or Metformin treatment differ. Firstly, comparative proteome and immunoblot analyses revealed that proteins promoting the G1/S cell cycle transition as well as downstream targets of the DREAM complex or pRb are downregulated upon E6/E7 silencing by RNAi. This indicates that the DREAM complex is active and thereby functioning in its role as a transcriptional repressor [55, 61, 62, 64]. In contrast, this regulation was not evident upon E6/E7 downregulation by Metformin. However, if proliferation arrest is E6/E7‐dependent, a downregulation of these cell cycle‐promoting genes would be expected, since growth inhibition via E6/E7 repression is linked to the restoration of DREAM and pRb activities [37, 61, 64]. Secondly, the regulation of cell cycle in HPV‐positive cancer cells differed between the two approaches, further supporting distinct mechanisms of growth inhibition. In line with the suppression of factors promoting G1/S transition, RNAi‐mediated E6/E7 repression led to a strong G1 arrest, as expected [37, 66]. In contrast, Metformin treatment did not lead to an appreciable G1 arrest, although the cells were also efficiently growth inhibited. Thirdly, we found that lower doses of Metformin, which do not downregulate E6/E7 levels, were still able to efficiently block the proliferation of HPV‐positive cancer cells. Collectively, these findings provide strong evidence that the downregulation of E6/E7 by Metformin is not essential for its anti‐proliferative effect in HPV‐positive cancer cells, which is even observable in the presence of E6/E7. Moreover, the discrepant regulation of proteins involved in proliferation control and the dissimilar cell cycle responses further corroborate that the mechanisms underlying growth inhibition by RNAi‐mediated E6/E7 repression or Metformin treatment differ.

Metformin is further discussed to be employed as an adjuvant drug in combination with different chemotherapeutic agents. Yet, controversial results have been reported in preclinical and clinical studies, describing agonistic, antagonistic, or no effects of Metformin on the efficacy of chemotherapeutics [15, 17, 24, 25]. We previously showed that pre‐treatment of HPV‐positive cancer cells with Metformin interferes with senescence induction by the chemotherapeutic agent Etoposide [33]. Treatment of proliferating cells with Etoposide leads to a pronounced G2/M cell cycle arrest and to an accumulation of cells in the S phase, where it exerts genotoxic effects, thereby inducing a senescence response [74, 75, 90]. In the present study, we found that Metformin induces efficient growth arrest of HPV‐positive cancer cells, likely by slowing down cell cycle progression in several cell cycle phases, as it did not lead to a pronounced accumulation of the cells in a specific cell cycle phase. Importantly, Metformin‐treated cells no longer exhibited the pronounced increase in S phase populations and G2/M arrest in response to Etoposide exposure, which is seen in proliferating cells. These findings indicate that the interference with Etoposide‐induced senescence is a secondary effect of the Metformin‐induced inhibition of cellular growth and cell cycle progression, thereby protecting the cells against the genotoxic activity of Etoposide in the S phase of the cell cycle. This notion also suggests that similar effects could be mimicked by other growth‐inhibitory conditions, which is compatible with our finding that glucose deprivation also resulted in protection against Etoposide‐induced senescence. It is further noteworthy that these regulatory phenomena are not restricted to HPV‐positive cancer cells, as we found that Metformin pre‐treatment also interferes with Etoposide‐induced senescence in HPV‐negative cancer cells.

Cisplatin plays an important role in the clinical management of late‐stage and recurrent cervical cancer [5]. Numerous studies in different cancer models report that co‐treatment with Metformin can increase the anti‐cancer activity of Cisplatin both In Vitro and in the clinic [91, 92]. Notably, however, there are also several studies describing opposing effects in that co‐treatment with Metformin can decrease Cisplatin efficacy [93, 94, 95, 96, 97, 98, 99, 100]. In view of these discrepant results, we thoroughly analyzed the effect of this combinatorial treatment on the phenotype of HPV‐positive cancer cells using different treatment conditions. Interestingly, in cells that were simultaneously treated with Metformin and Cisplatin for 24 h and harvested immediately for further analyses, Metformin substantially decreased Cisplatin‐induced apoptosis. In strong contrast, pre‐treating cells with Metformin for 24 h and subsequently with both Metformin and Cisplatin for additional 24 h led to a clear increase of Cisplatin‐induced apoptosis, at least under certain Metformin concentrations. Thus, distinct variations in experimental conditions strongly affect the cellular response towards co‐treatment with Cisplatin and Metformin, and can even result in opposing effects on Cisplatin‐induced apoptosis.

Notably, the antagonistic effect of Metformin on Cisplatin‐induced apoptosis is only transient, as it was no longer observable when cells were harvested 24 h after drug removal. Furthermore, by addressing the long‐term phenotypic effects of co‐treatment with Cisplatin and Metformin upon drug removal, we observed cooperative growth‐inhibitory effects, regardless of whether the cells had been pre‐treated with Metformin. Senescence assays revealed that Cisplatin monotreatment can dose‐dependently induce senescence under both experimental conditions, in line with its pro‐senescent potential [10, 81]. The cooperative effect of co‐treating cells with Cisplatin and Metformin was more pronounced in Metformin‐pre‐treated cells and led to a substantially more efficient reduction of their colony formation capacities. It is also important to note that under this condition, the partial senescence response induced by lower Cisplatin concentrations (2.5 µM) was efficiently tightened by co‐treatment with 1 mM Metformin, whereas co‐treatment with 2.5 mM (HeLa) or 5 mM Metformin (SiHa) induced a phenotypic switch to cell death. Moreover, at higher Cisplatin concentrations (15 µM [HeLa] or 20 µM [SiHa]), which were strongly pro‐senescent under these treatment conditions, co‐treatment with Metformin induced a switch to cell death even at lower doses (1 mM). In contrast, in cells simultaneously treated with Metformin and Cisplatin (i.e., no pre‐treatment with Metformin), neither the enhancement of the senescence response nor a switch from senescence to cell death was detectable following the co‐treatment. Collectively, these results indicate that co‐treatment with Metformin and Cisplatin can lead to strong differences in cell fate decisions of HPV‐positive cancer cells, depending on precise experimental conditions.

Furthermore, we obtained strong experimental evidence that the regulation of p53 plays a major role in determining the different phenotypic outcomes under the above‐referenced treatment conditions. Cisplatin treatment alone led to a pronounced increase in the levels of total p53 and post‐translationally modified p53 forms. Importantly, concomitant silencing of p53 expression inhibited apoptosis in response to Cisplatin monotreatment as well as to co‐treatment with Cisplatin and Metformin, demonstrating a direct functional involvement of p53 in these processes. In line, the same anti‐apoptotic effects observed upon p53 repression were observed following downregulation of BID/t‐BID, which can promote p53‐dependent apoptosis as a downstream factor [101] and plays a key role for Cisplatin‐induced apoptosis in HPV‐positive cancer cells [39].

It is further important to note that Metformin monotreatment led to up‐ or downregulation of p53 levels in a dose‐ and time‐dependent manner. In this context, it is noteworthy that particularly after 24 h of Metformin treatment, p53 protein levels were strongly downregulated in both HeLa and SiHa cells. Since p53 is a critical mediator of Cisplatin‐induced apoptosis, this observation could explain the protective effect of Metformin against Cisplatin when the cells are simultaneously co‐treated for 24 h and harvested immediately. It will be interesting to decipher the exact mechanism of p53 downregulation by Metformin in future studies, which we found to be independent of HPV status. As we did not detect appreciable effects of Metformin on *TP53* mRNA levels, p53 downregulation could occur at the post‐transcriptional level. Indeed, Metformin has been found to exert post‐transcriptional effects, such as affecting mRNA translation [102, 103] or protein stability, which includes a study reporting destabilization of the p53 protein by Metformin in HeLa cells [104].

As another interesting observation in this context, we found that p53 levels did not always correlate with the strength of the apoptotic response under the tested experimental conditions, although our data show that p53 is clearly crucial for apoptosis induction upon Cisplatin and Metformin co‐treatment. A possible explanation for this observation could be provided by the accumulating evidence that the cellular response to p53 can be decisively influenced by the balance of additional pro‐ and anti‐apoptotic factors, which determine the cell's threshold for apoptosis induction [105]. Metformin, for instance, has been shown to decrease the levels of anti‐apoptotic members of the Bcl‐2 family [106, 107, 108, 109, 110]. It thus could be possible that these effects, in combination with pro‐apoptotic t‐BID, can tip the cells towards apoptosis after p53 activation [105, 111]. Additionally, p53 co‐factors which could influence cell fate decisions mediated by p53 [105, 112] might be affected by the broad spectrum of cellular effects exerted by Metformin treatment.

While our findings show that Metformin blocks tumor cell proliferation and can enhance the pro‐apoptotic effects of Cisplatin In Vitro, it is difficult to extrapolate its anti‐tumorigenic potential to the clinical setting from these results. One reason is that the Metformin concentrations employed in the literature (and also in our study) to achieve anti‐cancer effects In Vitro usually strongly exceed those attainable in the plasma of patients without causing intolerable side effects [17]. Of note, however, is that there are reports that Metformin could accumulate in tumor tissue [113, 114]. More pharmacokinetic studies on Metformin, particularly on its attainable concentration in tumors, could therefore be informative to define optimal scheduling for tumor therapy. If pharmacologically active Metformin doses are achievable in tumor cells of patients, our data would indicate that a concomitant application of Metformin could enhance the anti‐proliferative effects of Cisplatin, particularly following pre‐treatment with Metformin, and may also provide a Cisplatin dose de‐escalation strategy.

It is further important to note that In Vitro analyses assess the direct effects of Metformin on cancer cells. However, Metformin can also indirectly affect cancer growth and therefore, the activities of Metformin are likely even more complex. For example, Metformin can reduce glucose and insulin levels in the circulation, thereby lowering the concentrations of two factors, which may promote cancer growth [115, 116]. Moreover, Metformin has been found to exert pronounced effects on the tumor microenvironment. For example, Metformin can increase the anti‐tumorigenic activity of the immune system by affecting different immune cells [117, 118]. Metformin has also been reported to decrease the amount of hypoxia in tumors, including cervical cancer [119]. This latter finding is interesting since HPV‐positive cancer cells, as other cancer cells, exhibit increased resistance to chemo‐ and radiotherapy under hypoxic conditions [36, 39, 120, 121], and thus, Metformin may therapeutically sensitize cancer cells by increasing tissue oxygenation.

Collectively, our results reveal complex direct effects of Metformin alone and in combination with CT on the phenotype of HPV‐positive cancer cells, which are strongly dependent on individual experimental conditions. These findings could, at least partially, explain the discrepant results on Metformin reported in different experimental studies, including the contradictory reports on analyzing the effect of Metformin on the Cisplatin‐sensitivity of cancer cells. It is therefore difficult to predict the phenotypic responses of cancer cells towards Metformin treatment in different settings, which is further complicated by many variables that can additionally affect the response of cancer cells to Metformin In Vivo.

## Author Contributions

All authors contributed to the study conception and design. Material preparation, data collection, and analysis were performed by Alicia Avenhaus, Bianca J. Kuhn, Milica Velimirović, Tobias D. Strobel, Julia Bulkescher, Claudia Lohrey, Felix Hoppe‐Seyler, and Karin Hoppe‐Seyler. The first draft of the manuscript was written by Alicia Avenhaus, Milica Velimirović, Felix Hoppe‐Seyler, and Karin Hoppe‐Seyler. All authors have read and agreed to the published version of the manuscript.

## Conflicts of Interest

The authors declare no conflicts of interest.

## Supporting information

Supporting Information Figures revis.

Table S1 revis.

Table S2 revis.

Table S3 revis.

Table S4 revis.

## Data Availability

All data supporting the findings of this study are available from the corresponding author upon reasonable request. The mass spectrometry proteomics data have been deposited to the ProteomeXchange Consortium via the PRIDE [50] partner repository (https://www.ebi.ac.uk/pride/) with the data set identifiers PXD061861 (siE6/E7 versus siCtrl1) and PXD011095 (Metformin versus DMSO).
