# Supplementary material for: Pleiotropic Effects of Metformin on the Chemotherapy Response of HPV‐Positive Cancer Cells
Source: J Med Virol. 2025 Jun 16;97(6):e70434. doi: 10.1002/jmv.70434 (PMC12169209; doi:10.1002/jmv.70434)
Supplement: Supplementary file 1 — Supporting Information Figures revis. [file JMV-97-e70434-s001.pdf]

Supporting Information: Figure S1

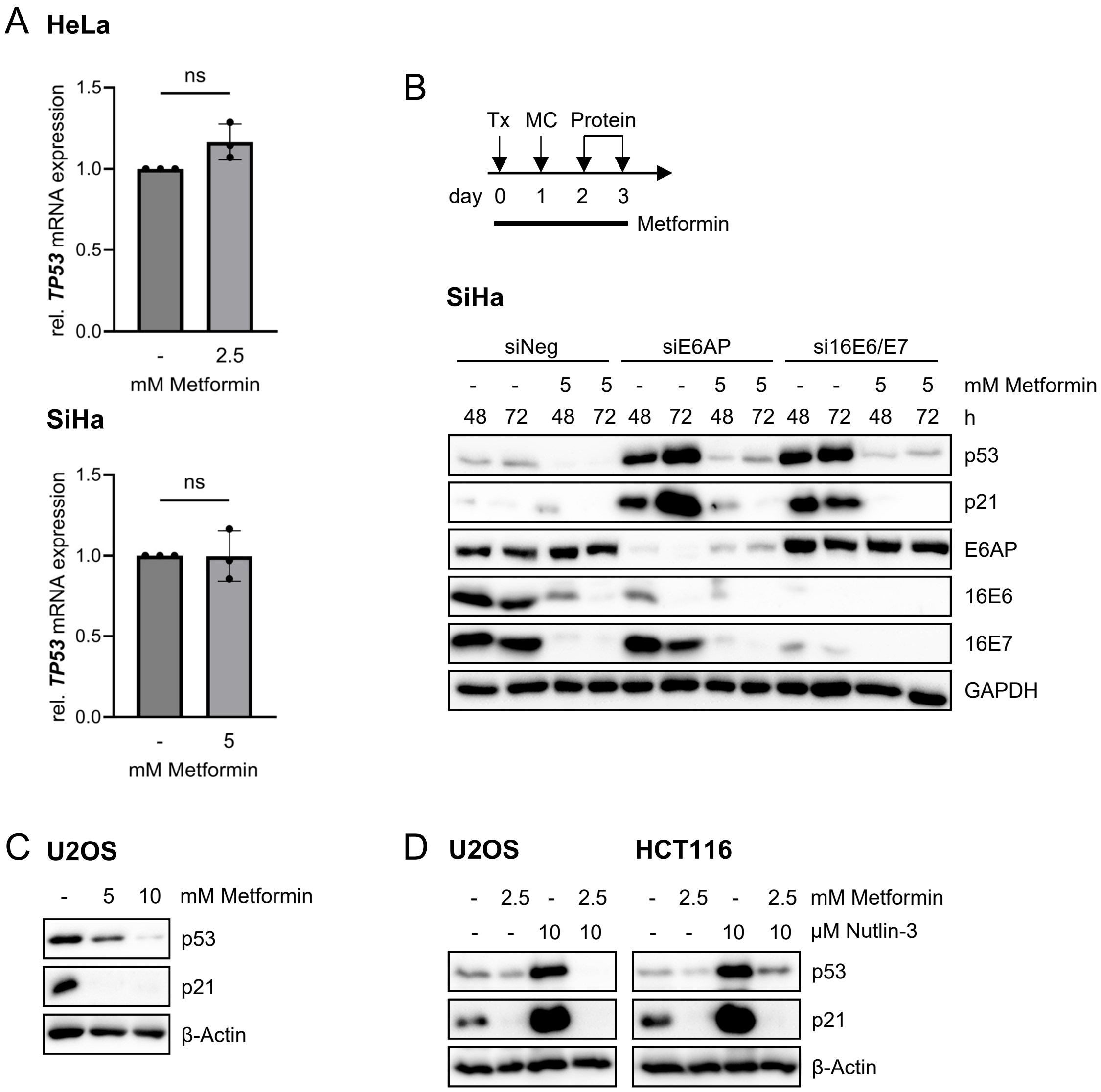

Figure S1: Reduction of p53 protein levels by Metformin. (A) HeLa or SiHa cells were treated with the indicated concentrations of Metformin for 24 h. qRT-PCR analyses of *TP53* mRNA levels were conducted and are shown as mean relative expression ± SD (n = 3), with the untreated control set to 1. Statistically significant differences were calculated using a two-sided unpaired t-test. ns, not significant. rel, relative. **(B)** Upper panel: Treatment scheme; SiHa cells were transfected with siE6AP, si16E6/E7, or control siRNA (siNeg) and simultaneously treated with 5 mM Metformin for 48 or 72 h, if indicated. Subsequently, cells were harvested for protein analyses. Tx, transfection; MC, medium change. Lower panel: Corresponding immunoblot analyses of p53, p21, E6AP, 16E6, 16E7, and GAPDH protein levels. **(C)** U2OS osteosarcoma cells were treated with the indicated concentrations of Metformin for 24 h and examined by immunoblot for p53, p21, and β-Actin protein levels. **(D)** U2OS or HCT116 colon cancer cells were treated with the indicated concentrations of Metformin for 48 h. Nutlin-3 or DMSO (- , solvent control) were added for the last 24 h of treatment. Protein levels of p53, p21, and β-Actin were measured by immunoblot analyses.

Supporting Information: Figure S2

A **SiHa**

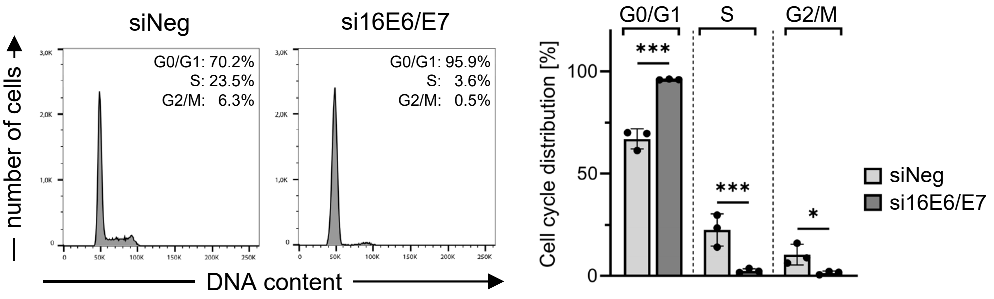

B **SiHa**

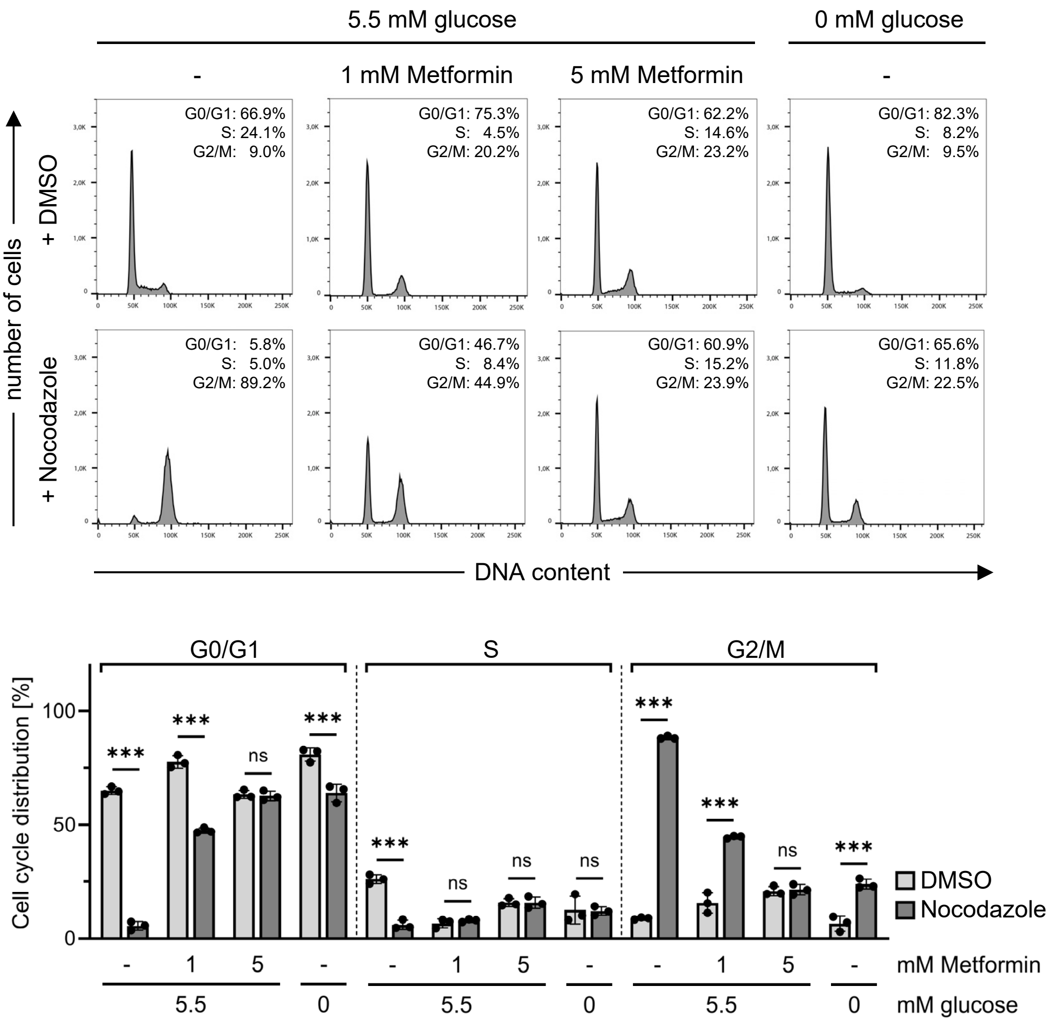

Figure S2: Metformin blocks proliferation of SiHa cells without pronounced accumulation of cells in a specific cell cycle phase. (A) SiHa cells were transfected with si16E6/E7 or control siRNA (siNeg), and grown for 72 h. Cell cycle profiles were analyzed by flow cytometry. Left panel: Results of one representative experiment with corresponding quantifications of the cell populations across the individual cell cycle phases. Right panel: Cell cycle distribution measured in three independent experiments, shown as mean percentages ± SD. Statistically significant differences were calculated using two-way ANOVA with Tukey's test for multiple comparisons. Comparisons between siNeg and si16E6/E7 within each cell cycle phase are indicated. * $p \leq 0.05$, *** $p \leq 0.001$. **(B)** SiHa cells were treated with the indicated concentrations of Metformin or cultivated in medium containing no glucose (0 mM) for 48 h. Nocodazole at a final concentration of 0.1 µg/mL or DMSO (solvent control) were added for the last 24 h of treatment. Cell cycle profiles were analyzed by flow cytometry. Upper panel: Results of one representative experiment with corresponding quantifications of the cell populations across the individual cell cycle phases. Lower panel: Cell cycle distribution measured in three independent experiments, shown as mean percentages ± SD. Statistically significant differences were calculated using two-way ANOVA with Tukey's test for multiple comparisons. Comparisons between DMSO and Nocodazole for the same cell cycle phase within each condition are indicated. ns, not significant, *** $p \leq 0.001$.

Supporting Information: Figure S3

SiHa

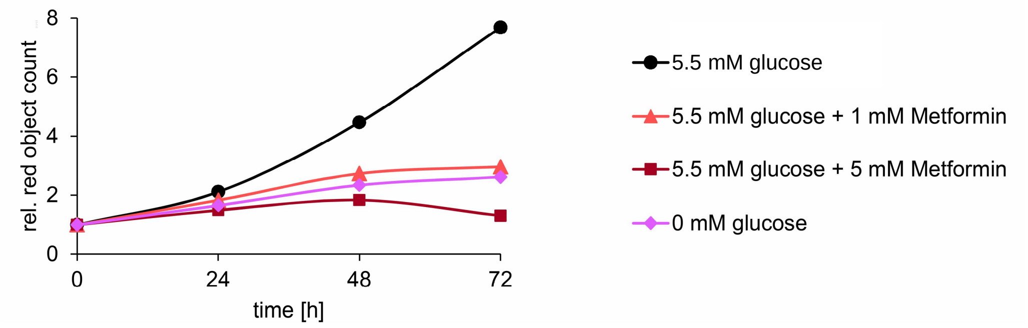

Figure S3: Metformin treatment or glucose deprivation inhibits the proliferation of SiHa cells. SiHa-mKate2 cells were treated 24 h after seeding (time point 0) with the indicated concentrations of Metformin or cultivated in medium containing no glucose (0 mM) for 72 h. Cells were imaged by the Incucyte® S3 live-cell imaging system. Red objects (cell counts) were quantified with the Incucyte® software package and are shown relative to the number of red objects at time point 0 (set to 1). rel, relative.

Supporting Information: Figure S4

HeLa

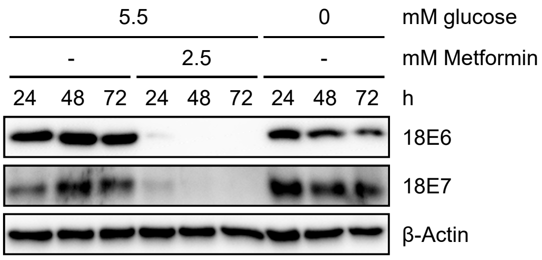

Figure S4: Glucose deprivation does not appreciably affect viral E6/E7 expression in HeLa cells. HeLa cells were treated with the indicated concentrations of Metformin or cultivated in medium containing no glucose (0 mM) for up to 72 h. Protein levels of 18E6, 18E7, and β-Actin were measured by immunoblot analyses.

Supporting Information: Figure S5

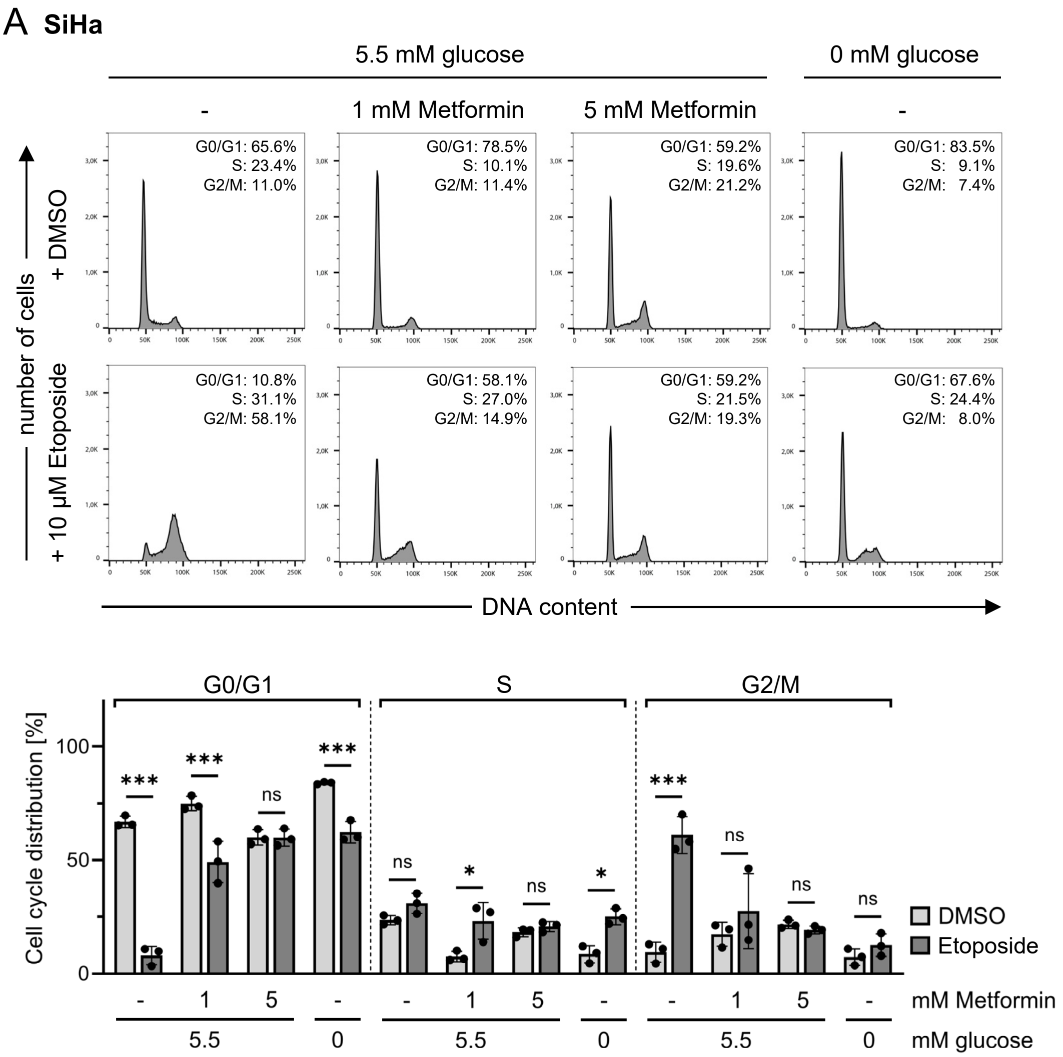

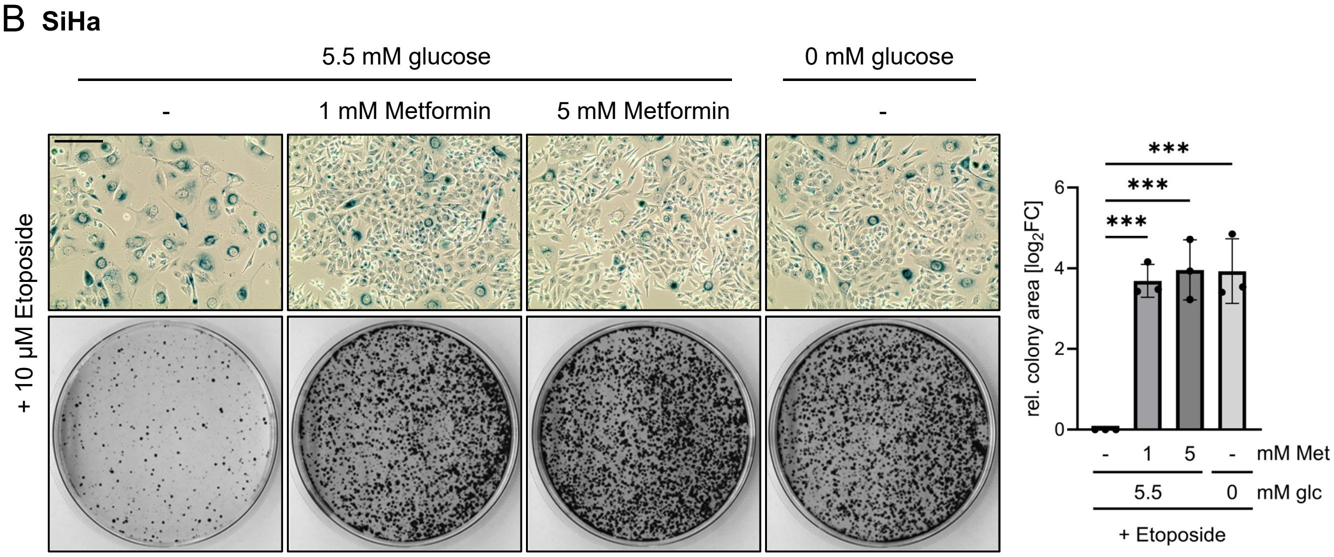

Figure S5: *See figure legend on the next page.*

Figure S5: Metformin treatment or glucose deprivation protects SiHa cells from the pro-senescent effects of Etoposide. SiHa cells were treated following the treatment scheme depicted in Figure 3A. **(A)** Cell cycle profiles were analyzed by flow cytometry. Upper panel: Results of one representative experiment with corresponding quantifications of the cell populations across the individual cell cycle phases. Lower panel: Cell cycle distribution measured in three independent experiments, shown as mean percentages ± SD. Statistically significant differences were calculated using two-way ANOVA with Tukey's test for multiple comparisons. Comparisons between DMSO and Etoposide for the same cell cycle phase within each condition are indicated. ns, not significant, * $p \leq 0.05$, *** $p \leq 0.001$. **(B)** Left panel: Corresponding senescence assays (upper panels; SA-β-Gal staining, blue; scale bar: 200 µm) and CFAs (lower panels). Right panel: Quantification of colony areas from three independent CFA experiments. Log_2-transformed fold changes (log_2FC) of mean colony area ± SD are shown. Statistically significant differences were calculated using one-way ANOVA with Tukey's test for multiple comparisons. Comparisons between Etoposide-treated cells ($log_2FC = 0$) and each other condition are indicated. *** $p \leq 0.001$. rel, relative; Met, Metformin; glc, glucose.

Supporting Information: Figure S6

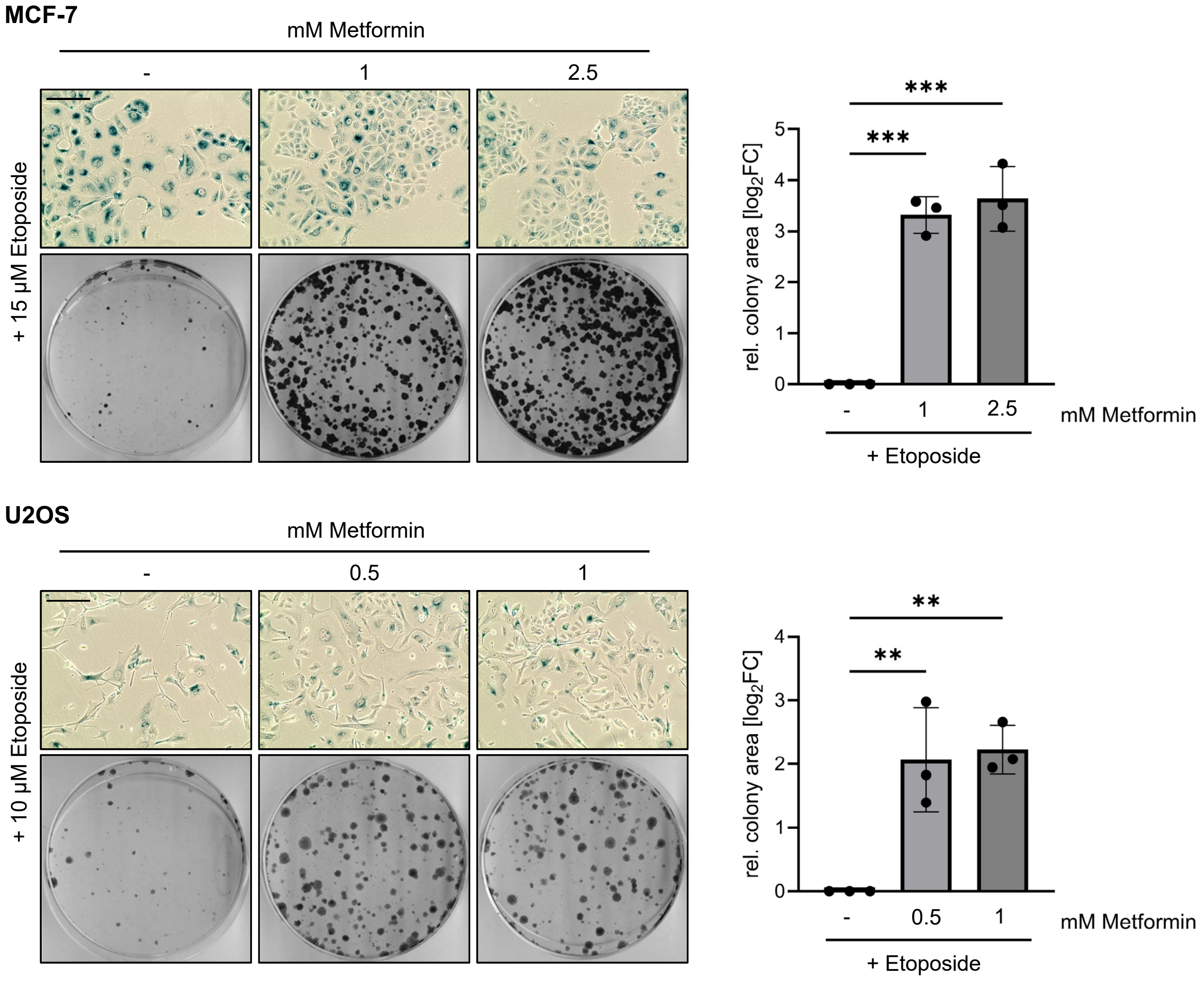

Figure S6: Metformin treatment can protect HPV-negative cancer cells from Etoposide-induced senescence. MCF-7 breast carcinoma or U2OS osteosarcoma cells were treated with the indicated concentrations of Metformin for 72 h. Etoposide was added for the last 24 h of treatment. Subsequently, cells were split and further cultivated in drug-free medium. Left panels: Senescence assays, performed on day 4 after splitting (upper panels for each cell line; SA-β-Gal staining, blue; scale bar: 200 µm) or CFAs, performed on day 12 after splitting (lower panels for each cell line). Right panels: Quantification of colony areas from three independent CFA experiments. Log_2-transformed fold changes (log_2FC) of mean colony area ± SD are shown. Statistically significant differences were calculated using one-way ANOVA with Tukey's test for multiple comparisons. Comparisons between Etoposide-treated cells ($log_2FC = 0$) and each other condition are indicated. ** $p \le 0.01$, *** $p \le 0.001$. rel, relative.

Supporting Information: Figure S7

HeLa

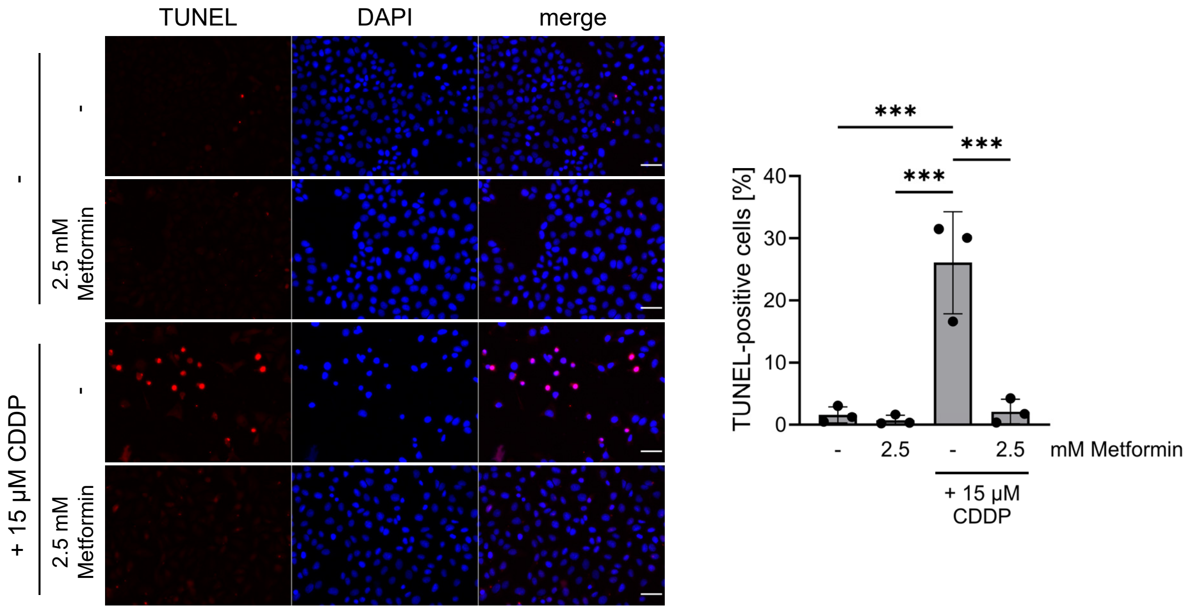

SiHa

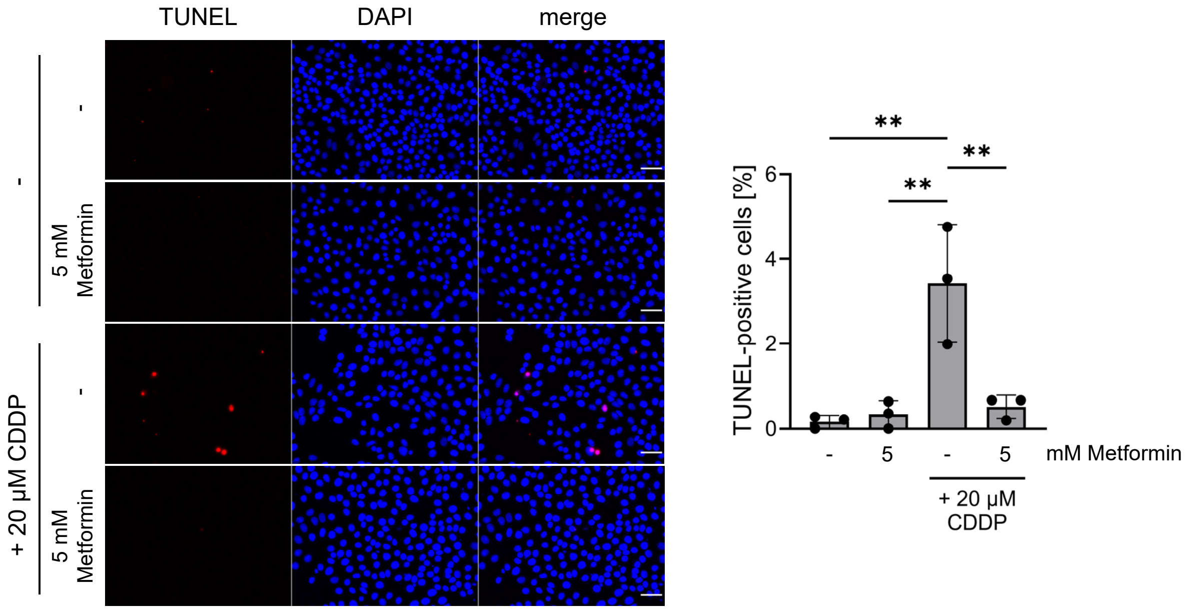

Figure S7: Simultaneous treatment with Metformin and Cisplatin protects HPV-positive cancer cells from Cisplatin-induced apoptosis. HeLa or SiHa cells were treated following the treatment scheme in Figure 4A and harvested for TUNEL assays at the end of treatment. Left panels: TUNEL analyses of HeLa or SiHa cells (scale bar: 50 µm). Right panels: TUNEL-positive (apoptotic) cells were quantified relative to the number of DAPI-stained nuclei. Mean percentages ± SD (n = 15 fields of view, each containing ≥ 50 cells, analyzed from three independent experiments) are shown. Statistically significant differences were calculated using one-way ANOVA with Tukey's test for multiple comparisons. Comparisons between CDDP-treated cells and each other condition are indicated. ** $p \leq 0.01$, *** $p \leq 0.001$.

Supporting Information: Figure S8

A **HeLa**

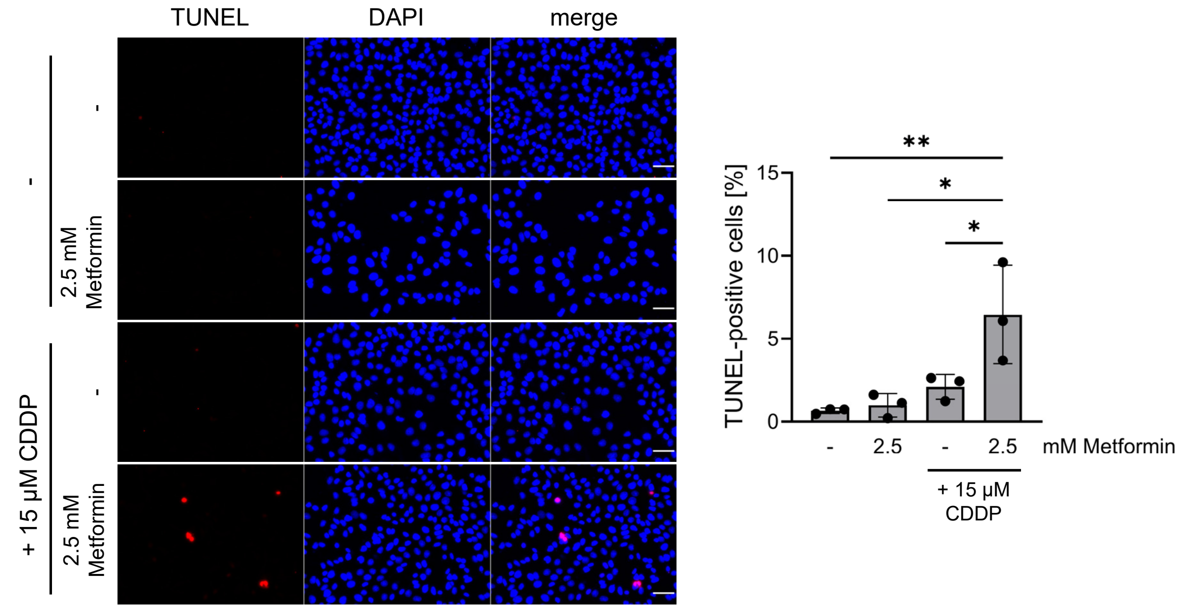

SiHa

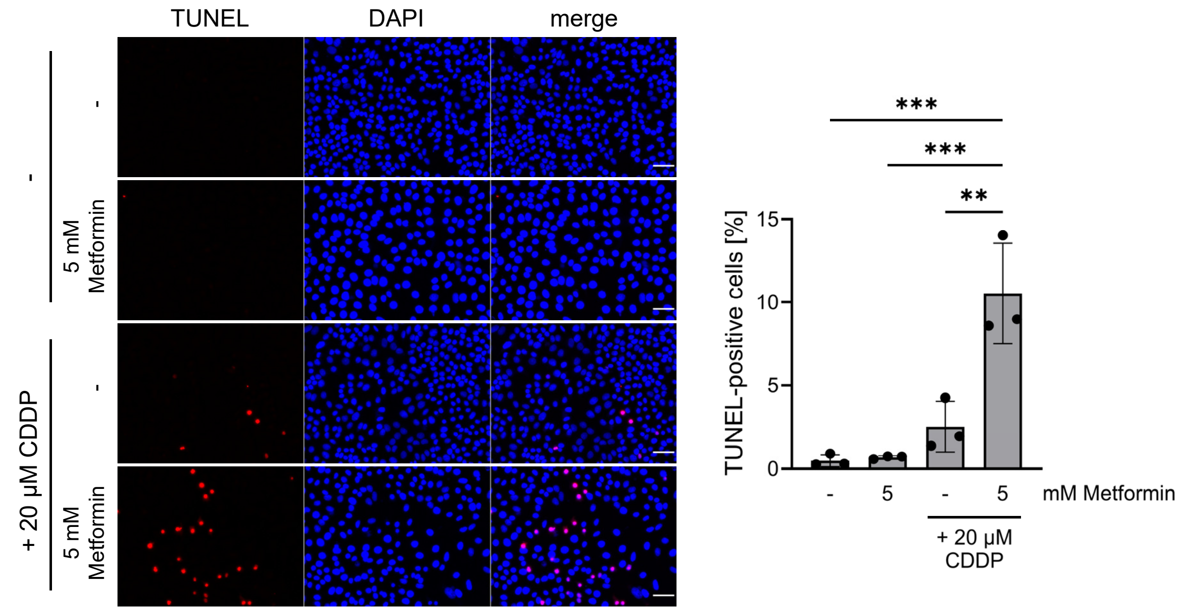

B **HeLa**

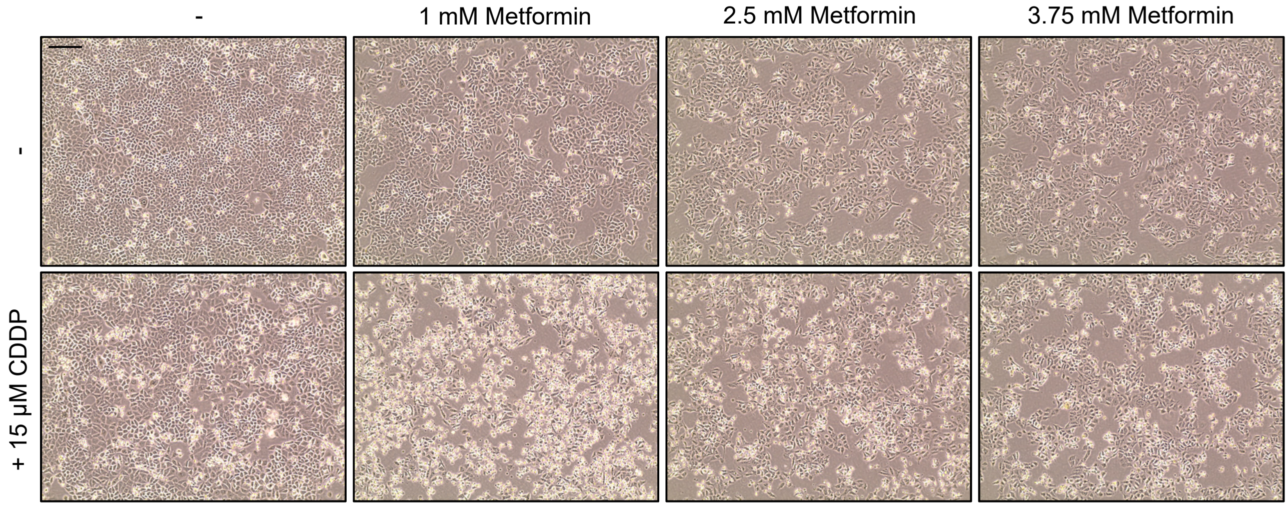

Figure S8: *See figure legend on the next page.*

Figure S8: Pre-treatment with Metformin can enhance the pro-apoptotic effects of Cisplatin in HPV-positive cancer cells. (A) HeLa or SiHa cells were treated following the treatment scheme in Figure 4B and harvested for TUNEL assays at the end of treatment. Left panels: TUNEL analyses of HeLa or SiHa cells (scale bar: 50 µm). Right panels: TUNEL-positive (apoptotic) cells were quantified relative to the number of DAPI-stained nuclei. Mean percentages ± SD (n = 15 fields of view, each containing ≥ 50 cells, analyzed from three independent experiments) are shown. Statistically significant differences were calculated using one-way ANOVA with Tukey's test for multiple comparisons. Comparisons between Metformin + CDDP-treated cells and each other condition are indicated. * $p \leq 0.05$, ** $p \leq 0.01$, *** $p \leq 0.001$. **(B)** HeLa cells were treated following the treatment scheme in Figure 4B. Light microscopy images taken at the time of protein harvest with an EVOSxl Core Cell Imaging System at 10× magnification are shown (scale bar: 200 µm).

Supporting Information: Figure S9

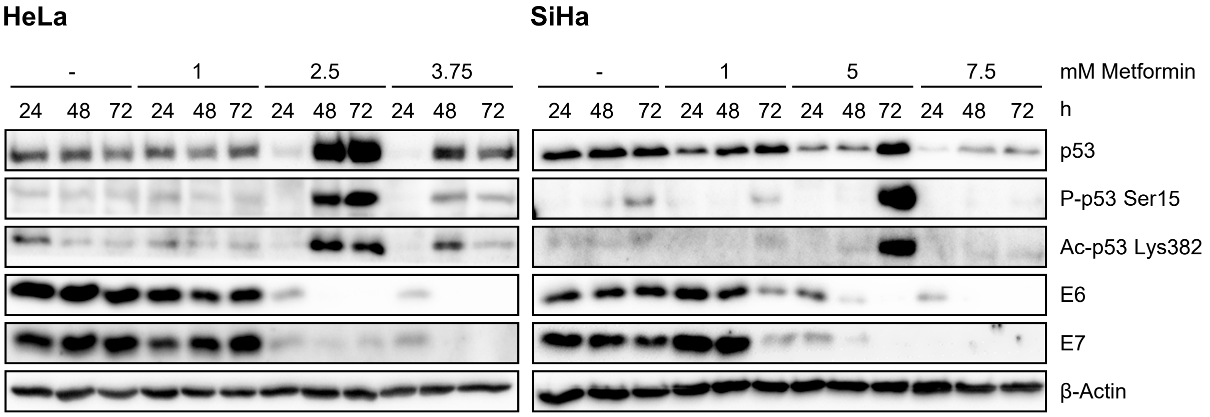

Figure S9: Metformin can decrease or increase p53 levels in HPV-positive cancer cells in a time- and dose-dependent manner. HeLa or SiHa cells were treated with the indicated concentrations of Metformin for up to 72 h. Protein levels of p53, P-p53 Ser15, Ac-p53 Lys382, E6, E7, and β-Actin were measured by immunoblot analyses.

Supporting Information: Figure S10

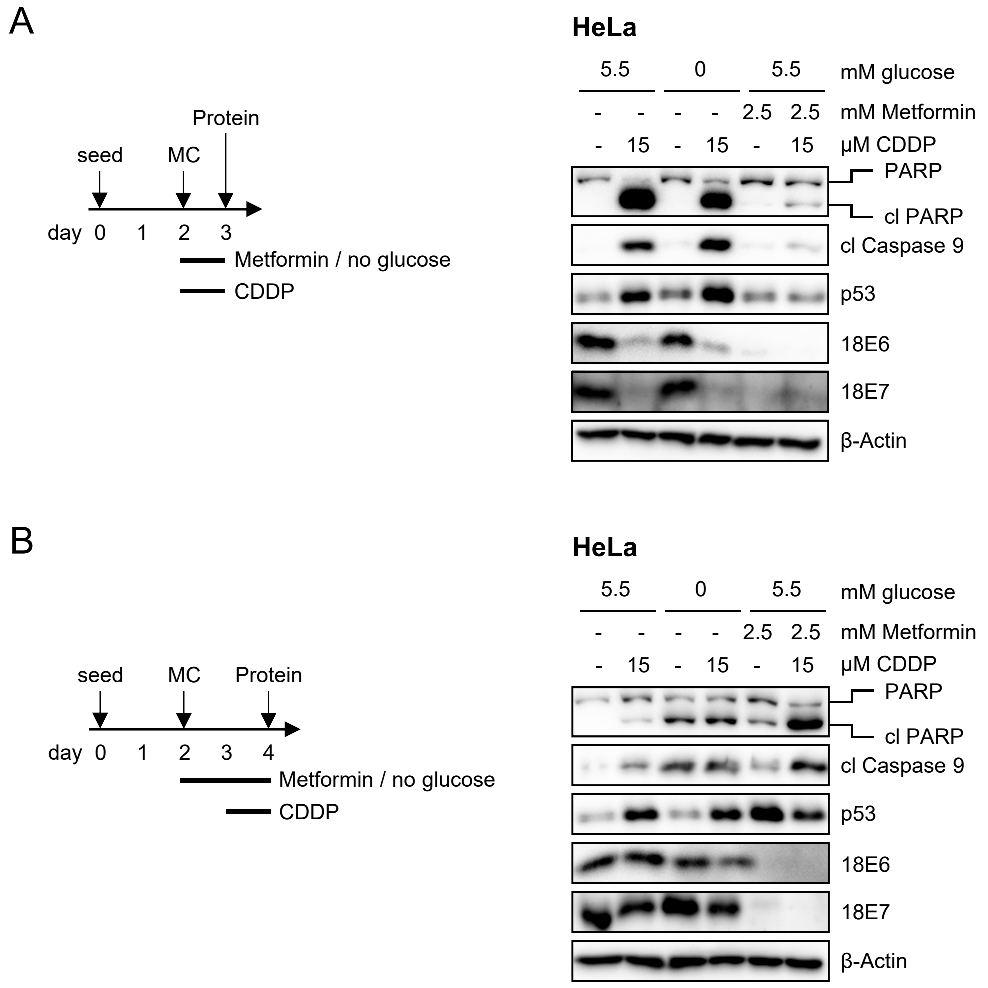

Figure S10: Glucose deprivation does not mimic the effects of Metformin on Cisplatin-induced apoptosis in HeLa cells. (A) Left panel: Treatment scheme; HeLa cells were treated with the indicated concentrations of Metformin and/or Cisplatin (CDDP) and/or were cultivated in medium containing no glucose (0 mM) for 24 h. Subsequently, cells were harvested for protein analyses. MC, medium change. Right panel: Corresponding immunoblot analyses of PARP, cleaved (cl) PARP, cl Caspase 9, p53, 18E6, 18E7, and β-Actin protein levels. **(B)** Left panel: Treatment scheme; HeLa cells were treated with the indicated concentrations of Metformin or cultivated in medium containing no glucose (0 mM) for 48 h. CDDP was added for the last 24 h of treatment, if indicated. Subsequently, cells were harvested for protein analyses. Right panel: Corresponding immunoblot analyses of PARP, cl PARP, cl Caspase 9, p53, 18E6, 18E7, and β-Actin protein levels.

Supporting Information: Figure S11

SiHa

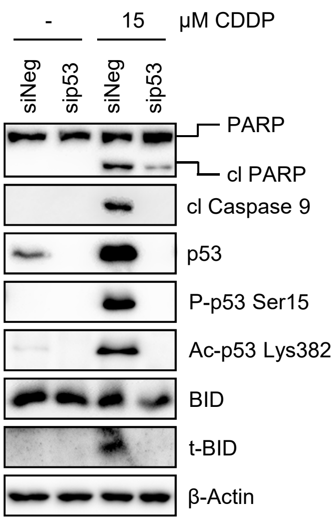

Figure S11: p53 is critical for Cisplatin-induced apoptosis in SiHa cells. SiHa cells were transfected with sip53 or control siRNA (siNeg). 48 h after transfection, the medium was changed and cells were treated with Cisplatin (CDDP) for 24 h. Protein levels of PARP, cleaved (cl) PARP, cl Caspase 9, p53, P-p53 Ser15, Ac-p53 Lys382, BID, t-BID, and β-Actin were measured by immunoblot analyses.

Supporting Information: Figure S12

HeLa

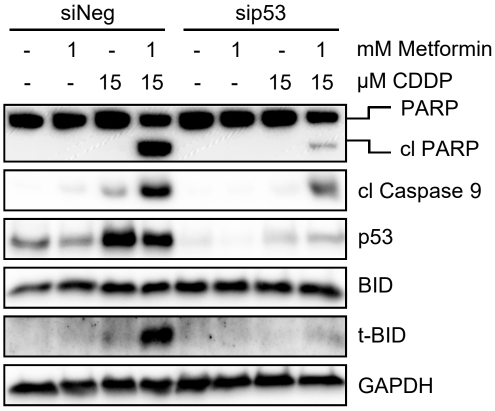

Figure S12: p53 is critical for the enhancing effects of 1 mM Metformin on Cisplatin-induced apoptosis in HeLa cells. HeLa cells were transfected with sip53 or control siRNA (siNeg) and treated with the indicated concentrations of Metformin and/or Cisplatin (CDDP) following the treatment scheme depicted in Figure 6. Protein levels of PARP, cleaved (cl) PARP, cl Caspase 9, p53, BID, t-BID, and GAPDH were measured by immunoblot analyses.

Supporting Information: Figure S13

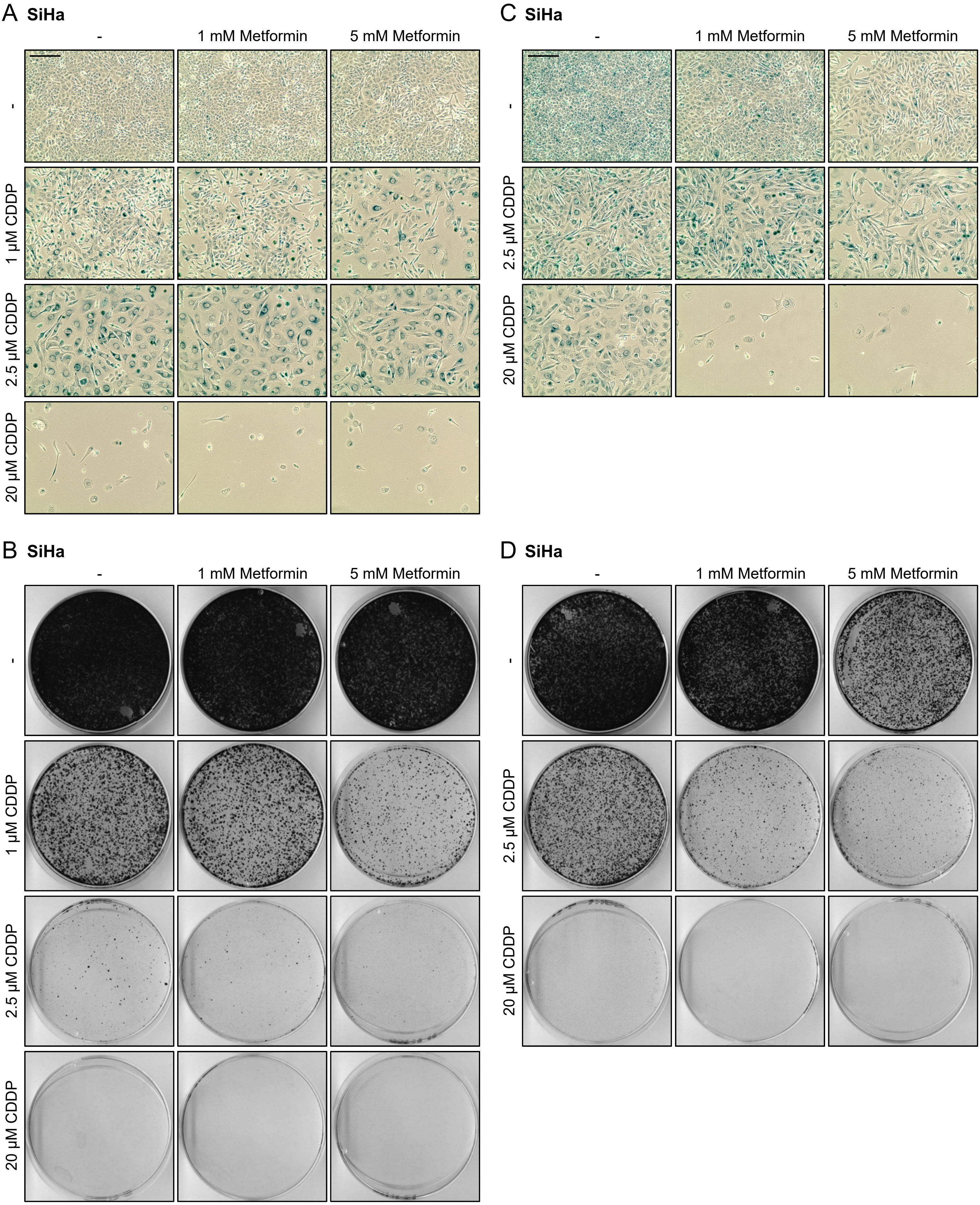

Figure S13: Metformin and Cisplatin can cooperate to inhibit long-term growth of SiHa cells. (A) Senescence assays (SA-β-Gal staining, blue; scale bar: 200 µm) and **(B)** CFAs of SiHa cells treated as depicted in Figure 7A. **(C)** Senescence assays (SA-β-Gal staining, blue; scale bar: 200 µm) and **(D)** CFAs of SiHa cells treated as depicted in Figure 7D.

Supporting Information: Figure S14

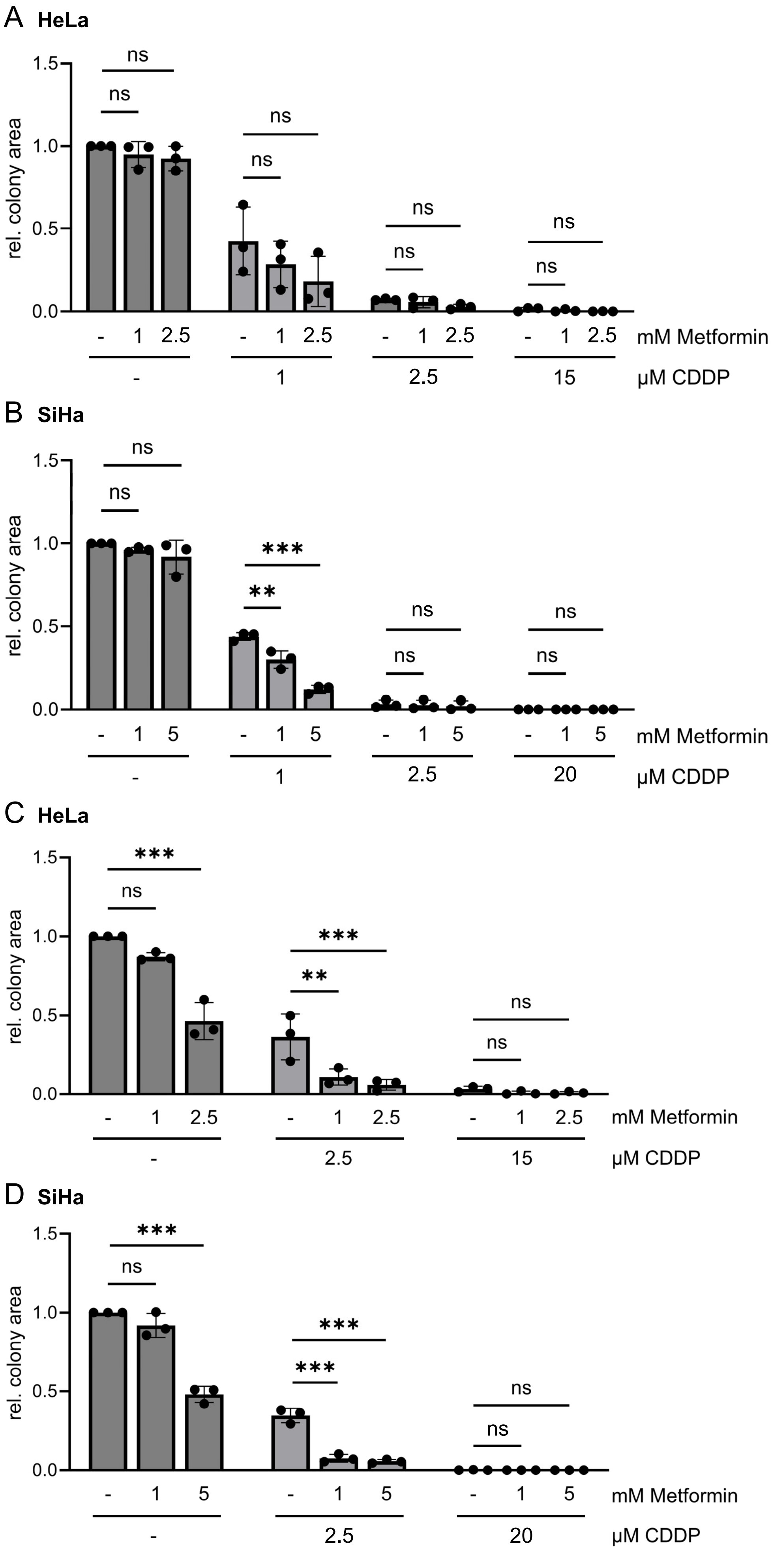

Figure S14: *See figure legend on the next page.*

Figure S14: Quantification of CFAs shown in Figures 7 and S13. Quantification of colony areas from three independent experiments corresponding to the representative CFAs shown in Figures 7C **(A)**, S13B **(B)**, 7F **(C)**, and S13D **(D)**. Data are presented as mean ± SD, with the untreated control set to 1. Statistically significant differences were calculated using two-way ANOVA with Tukey's test for multiple comparisons. Comparisons between untreated or CDDP-treated cells and the corresponding Metformin co-treated cells are indicated. ns, not significant, ** $p \leq 0.01$, *** $p \leq 0.001$. rel, relative.

Supporting Information: Figure S15

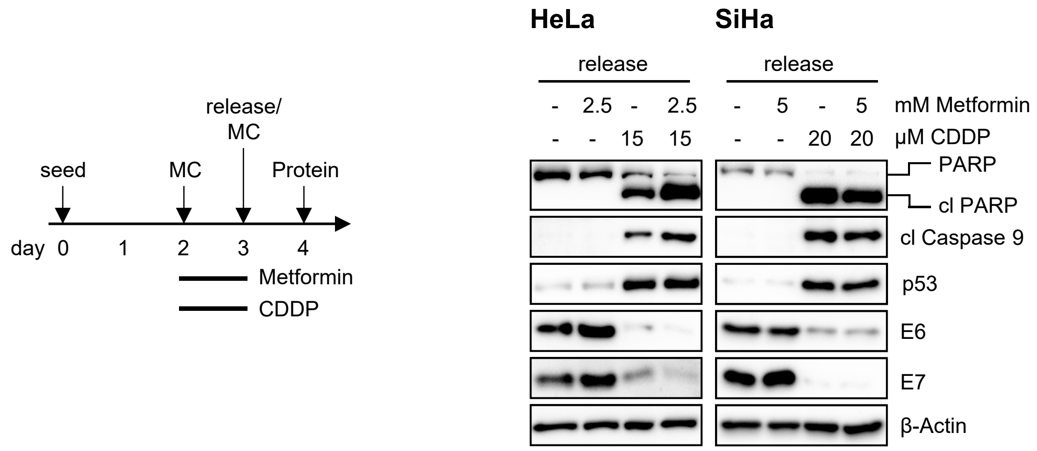

Figure S15: Increased expression of apoptosis markers in HPV-positive cancer cells 24 hours after release from co-treatment with Metformin and Cisplatin. Left panel: Treatment scheme; HeLa or SiHa cells were treated with the indicated concentrations of Metformin and/or Cisplatin (CDDP) for 24 h. Subsequently, the medium was replaced with drug-free medium (release), and the cells were cultivated for another 24 h before harvesting for protein analyses. MC, medium change. Right panel: Corresponding immunoblot analyses of PARP, cleaved (cl) PARP, cl Caspase 9, p53, E6, E7, and β-Actin protein levels.
