# Supplementary material for: Pleiotropic Effects of Metformin on the Chemotherapy Response of HPV‐Positive Cancer Cells
Source: J Med Virol. 2025 Jun 16;97(6):e70434. doi: 10.1002/jmv.70434 (PMC12169209; doi:10.1002/jmv.70434)
Supplement: Supplementary file 2 — Table S1 revis. [file JMV-97-e70434-s002.docx]

Supporting Information: Table S1

**Table S1: Densitometric quantification of immunoblots.** Upper rows show the ratio of net protein signal to the net loading control signal. Lower rows show values normalized to the sample highlighted in **blue** (if possible the first lane).

Figure 1A HeLa:

| **HeLa** | **siNeg vs siE6/E7** | | **untreated vs Metformin** | | | |
| --- | --- | --- | --- | --- | --- | --- |
| Lane → | **1** | **2** | **1** | **2** | **3** | **4** |
| **HPVE6** | **1.16** | 0.24 | **1.65** | 1.20 | 0.18 | 0.11 |
|  | **1.00** | 0.21 | **1.00** | 0.72 | 0.11 | 0.06 |

| **HeLa** | **siNeg vs siE6/E7** | | **untreated vs Metformin** | | | |
| --- | --- | --- | --- | --- | --- | --- |
| Lane → | **1** | **2** | **1** | **2** | **3** | **4** |
| **HPVE7** | **1.13** | 0.25 | **1.64** | 1.35 | 0.52 | 0.39 |
|  | **1.00** | 0.22 | **1.00** | 0.83 | 0.32 | 0.24 |

| **HeLa** | **siNeg vs siE6/E7** | | **untreated vs Metformin** | | | |
| --- | --- | --- | --- | --- | --- | --- |
| Lane → | **1** | **2** | **1** | **2** | **3** | **4** |
| **p53** | **0.82** | 1.63 | **1.33** | 1.31 | 0.39 | 0.14 |
|  | **1.00** | 2.00 | **1.00** | 0.99 | 0.29 | 0.11 |

| **HeLa** | **siNeg vs siE6/E7** | | **untreated vs Metformin** | | | |
| --- | --- | --- | --- | --- | --- | --- |
| Lane → | **1** | **2** | **1** | **2** | **3** | **4** |
| **p21** | **0.25** | 1.67 | **1.89** | 1.40 | 0.13 | 0.03 |
|  | **1.00** | 6.67 | **1.00** | 0.74 | 0.07 | 0.02 |

| **HeLa** | **siNeg vs siE6/E7** | | **untreated vs Metformin** | | | |
| --- | --- | --- | --- | --- | --- | --- |
| Lane → | **1** | **2** | **1** | **2** | **3** | **4** |
| **P-pRb** | **0.88** | 0.32 | **2.17** | 1.99 | 1.05 | 1.19 |
|  | **1.00** | 0.36 | **1.00** | 0.92 | 0.48 | 0.55 |

| **HeLa** | **siNeg vs siE6/E7** | | **untreated vs Metformin** | | | |
| --- | --- | --- | --- | --- | --- | --- |
| Lane → | **1** | **2** | **1** | **2** | **3** | **4** |
| **pRb** | **0.37** | 1.22 | **1.25** | 1.27 | 0.85 | 1.38 |
|  | **1.00** | 3.30 | **1.00** | 1.02 | 0.68 | 1.10 |

| **HeLa** | **siNeg vs siE6/E7** | | **untreated vs Metformin** | | | |
| --- | --- | --- | --- | --- | --- | --- |
| Lane → | **1** | **2** | **1** | **2** | **3** | **4** |
| **p130** | **0.53** | 1.55 | **1.80** | 1.44 | 0.64 | 0.39 |
|  | **1.00** | 2.93 | **1.00** | 0.80 | 0.36 | 0.22 |

| **HeLa** | **siNeg vs siE6/E7** | | **untreated vs Metformin** | | | |
| --- | --- | --- | --- | --- | --- | --- |
| Lane → | **1** | **2** | **1** | **2** | **3** | **4** |
| **Cyclin D1** | **0.56** | 1.16 | **1.23** | 0.47 | 0.02 | 0.01 |
|  | **1.00** | 2.10 | **1.00** | 0.38 | 0.02 | 0.01 |

Figure 1A SiHa:

| **SiHa** | **siNeg vs siE6/E7** | | **untreated vs Metformin** | | | |
| --- | --- | --- | --- | --- | --- | --- |
| Lane → | **1** | **2** | **1** | **2** | **3** | **4** |
| **HPVE6** | **1.20** | 0.06 | **1.03** | 0.73 | 0.34 | 0.17 |
|  | **1.00** | 0.05 | **1.00** | 0.70 | 0.33 | 0.17 |

| **SiHa** | **siNeg vs siE6/E7** | | **untreated vs Metformin** | | | |
| --- | --- | --- | --- | --- | --- | --- |
| Lane → | **1** | **2** | **1** | **2** | **3** | **4** |
| **HPVE7** | **1.42** | 0.15 | **1.14** | 1.21 | 0.26 | 0.09 |
|  | **1.00** | 0.11 | **1.00** | 1.06 | 0.22 | 0.08 |

| **SiHa** | **siNeg vs siE6/E7** | | **untreated vs Metformin** | | | |
| --- | --- | --- | --- | --- | --- | --- |
| Lane → | **1** | **2** | **1** | **2** | **3** | **4** |
| **p53** | **0.33** | 1.57 | **1.10** | 0.58 | 0.32 | 0.19 |
|  | **1.00** | 4.79 | **1.00** | 0.53 | 0.29 | 0.17 |

| **SiHa** | **siNeg vs siE6/E7** | | **untreated vs Metformin** | | | |
| --- | --- | --- | --- | --- | --- | --- |
| Lane → | **1** | **2** | **1** | **2** | **3** | **4** |
| **p21** | **0.29** | 1.56 | **0.68** | 0.88 | 0.93 | 0.41 |
|  | **1.00** | 5.45 | **1.00** | 1.30 | 1.37 | 0.60 |

| **SiHa** | **siNeg vs siE6/E7** | | **untreated vs Metformin** | | | |
| --- | --- | --- | --- | --- | --- | --- |
| Lane → | **1** | **2** | **1** | **2** | **3** | **4** |
| **P-pRb** | **1.33** | 0.26 | **1.14** | 1.20 | 0.55 | 0.41 |
|  | **1.00** | 0.20 | **1.00** | 1.05 | 0.48 | 0.36 |

| **SiHa** | **siNeg vs siE6/E7** | | **untreated vs Metformin** | | | |
| --- | --- | --- | --- | --- | --- | --- |
| Lane → | **1** | **2** | **1** | **2** | **3** | **4** |
| **pRb** | **0.83** | 1.17 | **1.02** | 1.14 | 1.05 | 1.26 |
|  | **1.00** | 1.41 | **1.00** | 1.12 | 1.03 | 1.24 |

| **SiHa** | **siNeg vs siE6/E7** | | **untreated vs Metformin** | | | |
| --- | --- | --- | --- | --- | --- | --- |
| Lane → | **1** | **2** | **1** | **2** | **3** | **4** |
| **p130** | **0.82** | 1.39 | **0.98** | 0.87 | 0.26 | 0.34 |
|  | **1.00** | 1.69 | **1.00** | 0.89 | 0.27 | 0.35 |

| **SiHa** | **siNeg vs siE6/E7** | | **untreated vs Metformin** | | | |
| --- | --- | --- | --- | --- | --- | --- |
| Lane → | **1** | **2** | **1** | **2** | **3** | **4** |
| **Cyclin D1** | **0.15** | 1.15 | **1.01** | 0.29 | 0.14 | 0.03 |
|  | **1.00** | 7.43 | **1.00** | 0.29 | 0.14 | 0.03 |

Figure 1C HeLa:

| **HeLa** | **siNeg vs siE6/E7** | | **untreated vs Metformin** | | | |
| --- | --- | --- | --- | --- | --- | --- |
| Lane → | **1** | **2** | **1** | **2** | **3** | **4** |
| **B-MYB** | **1.12** | 0.21 | **1.41** | 1.15 | 1.11 | 0.84 |
|  | **1.00** | 0.19 | **1.00** | 0.82 | 0.79 | 0.60 |

| **HeLa** | **siNeg vs siE6/E7** | | **untreated vs Metformin** | | | |
| --- | --- | --- | --- | --- | --- | --- |
| Lane → | **1** | **2** | **1** | **2** | **3** | **4** |
| **FOXM1** | **0.99** | 0.15 | **1.14** | 1.17 | 0.75 | 0.46 |
|  | **1.00** | 0.15 | **1.00** | 1.03 | 0.66 | 0.41 |

| **HeLa** | **siNeg vs siE6/E7** | | **untreated vs Metformin** | | | |
| --- | --- | --- | --- | --- | --- | --- |
| Lane → | **1** | **2** | **1** | **2** | **3** | **4** |
| **E2F1** | **0.81** | 0.22 | **1.25** | 0.58 | 0.78 | 0.88 |
|  | **1.00** | 0.27 | **1.00** | 0.46 | 0.63 | 0.70 |

| **HeLa** | **siNeg vs siE6/E7** | | **untreated vs Metformin** | | | |
| --- | --- | --- | --- | --- | --- | --- |
| Lane → | **1** | **2** | **1** | **2** | **3** | **4** |
| **Cyclin A** | **0.97** | 0.14 | **1.20** | 1.16 | 1.07 | 0.90 |
|  | **1.00** | 0.14 | **1.00** | 0.96 | 0.89 | 0.75 |

| **HeLa** | **siNeg vs siE6/E7** | | **untreated vs Metformin** | | | |
| --- | --- | --- | --- | --- | --- | --- |
| Lane → | **1** | **2** | **1** | **2** | **3** | **4** |
| **Cyclin B1** | **0.87** | 0.23 | **1.29** | 1.17 | 1.00 | 0.81 |
|  | **1.00** | 0.27 | **1.00** | 0.91 | 0.77 | 0.63 |

| **HeLa** | **siNeg vs siE6/E7** | | **untreated vs Metformin** | | | |
| --- | --- | --- | --- | --- | --- | --- |
| Lane → | **1** | **2** | **1** | **2** | **3** | **4** |
| **Cyclin B2** | **1.17** | 0.14 | **0.96** | 1.04 | 0.70 | 0.41 |
|  | **1.00** | 0.12 | **1.00** | 1.09 | 0.73 | 0.43 |

| **HeLa** | **siNeg vs siE6/E7** | | **untreated vs Metformin** | | | |
| --- | --- | --- | --- | --- | --- | --- |
| Lane → | **1** | **2** | **1** | **2** | **3** | **4** |
| **CDC2** | **0.86** | 0.13 | **1.50** | 1.35 | 1.23 | 1.02 |
|  | **1.00** | 0.15 | **1.00** | 0.90 | 0.82 | 0.68 |

| **HeLa** | **siNeg vs siE6/E7** | | **untreated vs Metformin** | | | |
| --- | --- | --- | --- | --- | --- | --- |
| Lane → | **1** | **2** | **1** | **2** | **3** | **4** |
| **CDK2** | **0.53** | 0.20 | **1.03** | 0.86 | 0.87 | 0.78 |
|  | **1.00** | 0.37 | **1.00** | 0.84 | 0.85 | 0.76 |

| **HeLa** | **siNeg vs siE6/E7** | | **untreated vs Metformin** | | | |
| --- | --- | --- | --- | --- | --- | --- |
| Lane → | **1** | **2** | **1** | **2** | **3** | **4** |
| **CKS1** | **1.05** | 0.14 | **483.81** | 340.66 | 275.34 | 120.36 |
|  | **1.00** | 0.13 | **1.00** | 0.70 | 0.57 | 0.25 |

| **HeLa** | **siNeg vs siE6/E7** | | **untreated vs Metformin** | | | |
| --- | --- | --- | --- | --- | --- | --- |
| Lane → | **1** | **2** | **1** | **2** | **3** | **4** |
| **RRM1** | **0.97** | 0.40 | **1.06** | 0.73 | 0.64 | 0.92 |
|  | **1.00** | 0.41 | **1.00** | 0.68 | 0.60 | 0.86 |

| **HeLa** | **siNeg vs siE6/E7** | | **untreated vs Metformin** | | | |
| --- | --- | --- | --- | --- | --- | --- |
| Lane → | **1** | **2** | **1** | **2** | **3** | **4** |
| **RRM2** | **0.55** | 0.08 | **1.05** | 0.97 | 1.06 | 0.78 |
|  | **1.00** | 0.15 | **1.00** | 0.93 | 1.01 | 0.74 |

| **HeLa** | **siNeg vs siE6/E7** | | **untreated vs Metformin** | | | |
| --- | --- | --- | --- | --- | --- | --- |
| Lane → | **1** | **2** | **1** | **2** | **3** | **4** |
| **PCNA** | **0.93** | 0.52 | **0.99** | 0.93 | 0.93 | 0.96 |
|  | **1.00** | 0.56 | **1.00** | 0.93 | 0.93 | 0.96 |

| **HeLa** | **siNeg vs siE6/E7** | | **untreated vs Metformin** | | | |
| --- | --- | --- | --- | --- | --- | --- |
| Lane → | **1** | **2** | **1** | **2** | **3** | **4** |
| **PLK1** | **1.03** | 0.13 | **1.80** | 1.73 | 1.47 | 1.30 |
|  | **1.00** | 0.13 | **1.00** | 0.96 | 0.82 | 0.73 |

Figure 1C SiHa:

| **SiHa** | **siNeg vs siE6/E7** | | **untreated vs Metformin** | | | |
| --- | --- | --- | --- | --- | --- | --- |
| Lane → | **1** | **2** | **1** | **2** | **3** | **4** |
| **B-MYB** | **1.75** | 0.02 | **2.24** | 1.37 | 1.23 | 1.24 |
|  | **1.00** | 0.01 | **1.00** | 0.61 | 0.55 | 0.55 |

| **SiHa** | **siNeg vs siE6/E7** | | **untreated vs Metformin** | | | |
| --- | --- | --- | --- | --- | --- | --- |
| Lane → | **1** | **2** | **1** | **2** | **3** | **4** |
| **FOXM1** | **2.33** | 0.05 | **1.66** | 1.27 | 1.19 | 1.16 |
|  | **1.00** | 0.02 | **1.00** | 0.76 | 0.71 | 0.70 |

| **SiHa** | **siNeg vs siE6/E7** | | **untreated vs Metformin** | | | |
| --- | --- | --- | --- | --- | --- | --- |
| Lane → | **1** | **2** | **1** | **2** | **3** | **4** |
| **E2F1** | **1.49** | 0.01 | **1.87** | 1.36 | 1.45 | 1.37 |
|  | **1.00** | 0.01 | **1.00** | 0.73 | 0.77 | 0.73 |

| **SiHa** | **siNeg vs siE6/E7** | | **untreated vs Metformin** | | | |
| --- | --- | --- | --- | --- | --- | --- |
| Lane → | **1** | **2** | **1** | **2** | **3** | **4** |
| **Cyclin A** | **1.19** | 0.33 | **1.13** | 1.06 | 0.94 | 0.97 |
|  | **1.00** | 0.28 | **1.00** | 0.94 | 0.83 | 0.86 |

| **SiHa** | **siNeg vs siE6/E7** | | **untreated vs Metformin** | | | |
| --- | --- | --- | --- | --- | --- | --- |
| Lane → | **1** | **2** | **1** | **2** | **3** | **4** |
| **Cyclin B1** | **1.60** | 0.07 | **1.68** | 1.45 | 1.20 | 1.31 |
|  | **1.00** | 0.05 | **1.00** | 0.86 | 0.71 | 0.78 |

| **SiHa** | **siNeg vs siE6/E7** | | **untreated vs Metformin** | | | |
| --- | --- | --- | --- | --- | --- | --- |
| Lane → | **1** | **2** | **1** | **2** | **3** | **4** |
| **Cyclin B2** | **2.21** | 0.19 | **1.25** | 0.93 | 0.88 | 0.80 |
|  | **1.00** | 0.08 | **1.00** | 0.75 | 0.71 | 0.64 |

| **SiHa** | **siNeg vs siE6/E7** | | **untreated vs Metformin** | | | |
| --- | --- | --- | --- | --- | --- | --- |
| Lane → | **1** | **2** | **1** | **2** | **3** | **4** |
| **CDC2** | **1.58** | 0.02 | **1.49** | 1.20 | 1.10 | 1.17 |
|  | **1.00** | 0.01 | **1.00** | 0.81 | 0.74 | 0.79 |

| **SiHa** | **siNeg vs siE6/E7** | | **untreated vs Metformin** | | | |
| --- | --- | --- | --- | --- | --- | --- |
| Lane → | **1** | **2** | **1** | **2** | **3** | **4** |
| **CDK2** | **1.74** | 0.25 | **1.43** | 1.10 | 0.96 | 0.99 |
|  | **1.00** | 0.14 | **1.00** | 0.77 | 0.67 | 0.69 |

| **SiHa** | **siNeg vs siE6/E7** | | **untreated vs Metformin** | | | |
| --- | --- | --- | --- | --- | --- | --- |
| Lane → | **1** | **2** | **1** | **2** | **3** | **4** |
| **CKS1** | **2.04** | 0.00 | **1.58** | 1.00 | 0.95 | 0.97 |
|  | **1.00** | 0.00 | **1.00** | 0.63 | 0.60 | 0.61 |

| **SiHa** | **siNeg vs siE6/E7** | | **untreated vs Metformin** | | | |
| --- | --- | --- | --- | --- | --- | --- |
| Lane → | **1** | **2** | **1** | **2** | **3** | **4** |
| **RRM1** | **1.26** | 0.08 | **1.17** | 0.67 | 0.93 | 0.59 |
|  | **1.00** | 0.06 | **1.00** | 0.58 | 0.80 | 0.51 |

| **SiHa** | **siNeg vs siE6/E7** | | **untreated vs Metformin** | | | |
| --- | --- | --- | --- | --- | --- | --- |
| Lane → | **1** | **2** | **1** | **2** | **3** | **4** |
| **RRM2** | **1.63** | 0.06 | **1.22** | 0.90 | 0.79 | 0.75 |
|  | **1.00** | 0.03 | **1.00** | 0.74 | 0.64 | 0.62 |

| **SiHa** | **siNeg vs siE6/E7** | | **untreated vs Metformin** | | | |
| --- | --- | --- | --- | --- | --- | --- |
| Lane → | **1** | **2** | **1** | **2** | **3** | **4** |
| **PCNA** | **1.56** | 0.60 | **0.93** | 0.84 | 0.88 | 1.01 |
|  | **1.00** | 0.38 | **1.00** | 0.91 | 0.95 | 1.09 |

| **SiHa** | **siNeg vs siE6/E7** | | **untreated vs Metformin** | | | |
| --- | --- | --- | --- | --- | --- | --- |
| Lane → | **1** | **2** | **1** | **2** | **3** | **4** |
| **PLK1** | **2.84** | 0.46 | **2.42** | 1.88 | 1.37 | 1.25 |
|  | **1.00** | 0.16 | **1.00** | 0.78 | 0.57 | 0.52 |

Figure 4A HeLa:

| **HeLa** |  | | | | | | | |
| --- | --- | --- | --- | --- | --- | --- | --- | --- |
| Lane → | **1** | **2** | **3** | **4** | **5** | **6** | **7** | **8** |
| **cl PARP** | 0.02 | 0.04 | 0.07 | 0.18 | **1.58** | 1.27 | 0.29 | 0.14 |
|  | 0.01 | 0.03 | 0.04 | 0.11 | **1.00** | 0.80 | 0.18 | 0.09 |

| **HeLa** |  | | | | | | | |
| --- | --- | --- | --- | --- | --- | --- | --- | --- |
| Lane → | **1** | **2** | **3** | **4** | **5** | **6** | **7** | **8** |
| **cl Caspase9** | 0.02 | 0.06 | 0.04 | 0.07 | **0.96** | 0.84 | 0.23 | 0.03 |
|  | 0.02 | 0.06 | 0.04 | 0.07 | **1.00** | 0.88 | 0.24 | 0.03 |

| **HeLa** |  | | | | | | | |
| --- | --- | --- | --- | --- | --- | --- | --- | --- |
| Lane → | **1** | **2** | **3** | **4** | **5** | **6** | **7** | **8** |
| **p53** | **0.28** | 0.36 | 0.40 | 0.30 | 0.82 | 0.62 | 0.39 | 0.19 |
|  | **1.00** | 1.26 | 1.41 | 1.06 | 2.88 | 2.20 | 1.37 | 0.65 |

| **HeLa** |  | | | | | | | |
| --- | --- | --- | --- | --- | --- | --- | --- | --- |
| Lane → | **1** | **2** | **3** | **4** | **5** | **6** | **7** | **8** |
| **BID** | **1.04** | 1.31 | 1.06 | 0.87 | 1.08 | 1.16 | 0.94 | 0.71 |
|  | **1.00** | 1.25 | 1.02 | 0.84 | 1.04 | 1.11 | 0.90 | 0.68 |

| **HeLa** |  | | | | | | | |
| --- | --- | --- | --- | --- | --- | --- | --- | --- |
| Lane → | **1** | **2** | **3** | **4** | **5** | **6** | **7** | **8** |
| **t-BID** | 0.00 | 0.01 | 0.01 | 0.03 | **1.44** | 0.41 | 0.02 | 0.00 |
|  | 0.00 | 0.00 | 0.01 | 0.02 | **1.00** | 0.28 | 0.01 | 0.00 |

| **HeLa** |  | | | | | | | |
| --- | --- | --- | --- | --- | --- | --- | --- | --- |
| Lane → | **1** | **2** | **3** | **4** | **5** | **6** | **7** | **8** |
| **HPVE6** | **1.08** | 0.53 | 0.19 | 0.17 | 0.19 | 0.19 | 0.01 | 0.00 |
|  | **1.00** | 0.49 | 0.18 | 0.16 | 0.17 | 0.17 | 0.01 | 0.00 |

| **HeLa** |  | | | | | | | |
| --- | --- | --- | --- | --- | --- | --- | --- | --- |
| Lane → | **1** | **2** | **3** | **4** | **5** | **6** | **7** | **8** |
| **HPVE7** | **1.07** | 1.22 | 0.22 | 0.11 | 0.11 | 0.18 | 0.17 | 0.09 |
|  | **1.00** | 1.14 | 0.21 | 0.10 | 0.10 | 0.17 | 0.16 | 0.09 |

Figure 4A SiHa:

| **SiHa** |  | | | | | | | | | |
| --- | --- | --- | --- | --- | --- | --- | --- | --- | --- | --- |
| Lane → | **1** | **2** | **3** | **4** | **5** | **6** | **7** | **8** | **9** | **10** |
| **cl PARP** | 0.14 | 0.16 | 0.12 | 0.09 | 0.06 | **1.18** | 0.61 | 0.05 | 0.11 | 0.17 |
|  | 0.12 | 0.13 | 0.10 | 0.07 | 0.05 | **1.00** | 0.52 | 0.04 | 0.09 | 0.14 |

| **SiHa** |  | | | | | | | | | |
| --- | --- | --- | --- | --- | --- | --- | --- | --- | --- | --- |
| Lane → | **1** | **2** | **3** | **4** | **5** | **6** | **7** | **8** | **9** | **10** |
| **cl Caspase9** | 0.05 | 0.09 | 0.13 | 0.16 | 0.14 | **0.88** | 0.17 | 0.12 | 0.14 | 0.14 |
|  | 0.06 | 0.10 | 0.15 | 0.19 | 0.15 | **1.00** | 0.20 | 0.14 | 0.16 | 0.16 |

| **SiHa** |  | | | | | | | | | |
| --- | --- | --- | --- | --- | --- | --- | --- | --- | --- | --- |
| Lane → | **1** | **2** | **3** | **4** | **5** | **6** | **7** | **8** | **9** | **10** |
| **p53** | **0.36** | 0.21 | 0.04 | 0.01 | 0.01 | 0.67 | 0.56 | 0.15 | 0.03 | 0.02 |
|  | **1.00** | 0.60 | 0.11 | 0.02 | 0.02 | 1.87 | 1.56 | 0.41 | 0.08 | 0.05 |

| **SiHa** |  | | | | | | | | | |
| --- | --- | --- | --- | --- | --- | --- | --- | --- | --- | --- |
| Lane → | **1** | **2** | **3** | **4** | **5** | **6** | **7** | **8** | **9** | **10** |
| **BID** | **1.30** | 1.56 | 1.73 | 1.65 | 1.35 | 1.10 | 1.17 | 1.40 | 1.45 | 1.41 |
|  | **1.00** | 1.20 | 1.33 | 1.27 | 1.04 | 0.85 | 0.90 | 1.08 | 1.11 | 1.08 |

| **SiHa** |  | | | | | | | | | |
| --- | --- | --- | --- | --- | --- | --- | --- | --- | --- | --- |
| Lane → | **1** | **2** | **3** | **4** | **5** | **6** | **7** | **8** | **9** | **10** |
| **t-BID** | 0.02 | 0.12 | 0.21 | 0.24 | 0.26 | **1.10** | 0.23 | 0.29 | 0.23 | 0.12 |
|  | 0.01 | 0.11 | 0.19 | 0.22 | 0.24 | **1.00** | 0.21 | 0.26 | 0.21 | 0.11 |

| **SiHa** |  | | | | | | | | | |
| --- | --- | --- | --- | --- | --- | --- | --- | --- | --- | --- |
| Lane → | **1** | **2** | **3** | **4** | **5** | **6** | **7** | **8** | **9** | **10** |
| **HPVE6** | **1.39** | 1.10 | 0.26 | 0.15 | 0.29 | 0.02 | 0.01 | 0.01 | 0.02 | 0.01 |
|  | **1.00** | 0.79 | 0.19 | 0.11 | 0.21 | 0.01 | 0.01 | 0.01 | 0.02 | 0.00 |

| **SiHa** |  | | | | | | | | | |
| --- | --- | --- | --- | --- | --- | --- | --- | --- | --- | --- |
| Lane → | **1** | **2** | **3** | **4** | **5** | **6** | **7** | **8** | **9** | **10** |
| **HPVE7** | **1.50** | 2.14 | 0.10 | 0.03 | 0.01 | 0.02 | 0.01 | 0.02 | 0.02 | 0.01 |
|  | **1.00** | 1.43 | 0.07 | 0.02 | 0.01 | 0.02 | 0.01 | 0.01 | 0.01 | 0.00 |

Figure 4B HeLa:

| **HeLa** |  | | | | | | | |
| --- | --- | --- | --- | --- | --- | --- | --- | --- |
| Lane → | **1** | **2** | **3** | **4** | **5** | **6** | **7** | **8** |
| **cl PARP** | 0.01 | 0.02 | 0.00 | 0.01 | **0.14** | 1.22 | 1.17 | 0.06 |
|  | 0.09 | 0.17 | 0.03 | 0.10 | **1.00** | 8.75 | 8.43 | 0.46 |

| **HeLa** |  | | | | | | | |
| --- | --- | --- | --- | --- | --- | --- | --- | --- |
| Lane → | **1** | **2** | **3** | **4** | **5** | **6** | **7** | **8** |
| **cl Caspase9** | 0.02 | 0.04 | 0.06 | 0.07 | **0.40** | 1.13 | 0.88 | 0.08 |
|  | 0.05 | 0.10 | 0.14 | 0.18 | **1.00** | 2.83 | 2.21 | 0.21 |

| **HeLa** |  | | | | | | | |
| --- | --- | --- | --- | --- | --- | --- | --- | --- |
| Lane → | **1** | **2** | **3** | **4** | **5** | **6** | **7** | **8** |
| **p53** | **0.36** | 0.43 | 1.82 | 1.13 | 1.56 | 0.61 | 0.93 | 0.02 |
|  | **1.00** | 1.20 | 5.12 | 3.17 | 4.38 | 1.71 | 2.62 | 0.06 |

| **HeLa** |  | | | | | | | |
| --- | --- | --- | --- | --- | --- | --- | --- | --- |
| Lane → | **1** | **2** | **3** | **4** | **5** | **6** | **7** | **8** |
| **BID** | **1.71** | 1.42 | 0.93 | 0.98 | 1.28 | 1.38 | 1.38 | 1.12 |
|  | **1.00** | 0.83 | 0.55 | 0.58 | 0.75 | 0.81 | 0.81 | 0.65 |

| **HeLa** |  | | | | | | | |
| --- | --- | --- | --- | --- | --- | --- | --- | --- |
| Lane → | **1** | **2** | **3** | **4** | **5** | **6** | **7** | **8** |
| **t-BID** | 0.03 | 0.05 | 0.08 | 0.04 | **0.37** | 1.09 | 0.91 | 0.01 |
|  | 0.07 | 0.15 | 0.20 | 0.11 | **1.00** | 2.94 | 2.47 | 0.02 |

| **HeLa** |  | | | | | | | |
| --- | --- | --- | --- | --- | --- | --- | --- | --- |
| Lane → | **1** | **2** | **3** | **4** | **5** | **6** | **7** | **8** |
| **HPVE6** | **2.23** | 1.33 | 0.01 | 0.01 | 0.95 | 0.01 | 0.00 | 0.00 |
|  | **1.00** | 0.60 | 0.00 | 0.01 | 0.43 | 0.01 | 0.00 | 0.00 |

| **HeLa** |  | | | | | | | |
| --- | --- | --- | --- | --- | --- | --- | --- | --- |
| Lane → | **1** | **2** | **3** | **4** | **5** | **6** | **7** | **8** |
| **HPVE7** | **1.76** | 1.62 | 0.26 | 0.17 | 0.87 | 0.38 | 0.06 | 0.06 |
|  | **1.00** | 0.92 | 0.15 | 0.09 | 0.49 | 0.22 | 0.04 | 0.03 |

Figure 4B SiHa:

| **SiHa** |  | | | | | | | | | |
| --- | --- | --- | --- | --- | --- | --- | --- | --- | --- | --- |
| Lane → | **1** | **2** | **3** | **4** | **5** | **6** | **7** | **8** | **9** | **10** |
| **cl PARP** | 0.07 | 0.10 | 0.23 | 0.15 | 0.11 | **0.39** | 0.28 | 1.04 | 0.62 | 0.33 |
|  | 0.17 | 0.27 | 0.60 | 0.39 | 0.29 | **1.00** | 0.73 | 2.69 | 1.60 | 0.85 |

| **SiHa** |  | | | | | | | | | |
| --- | --- | --- | --- | --- | --- | --- | --- | --- | --- | --- |
| Lane → | **1** | **2** | **3** | **4** | **5** | **6** | **7** | **8** | **9** | **10** |
| **cl Caspase9** | 0.00 | 0.00 | 0.02 | 0.04 | 0.03 | **0.02** | 0.03 | 0.86 | 0.36 | 0.12 |
|  | 0.05 | 0.20 | 0.77 | 2.21 | 1.29 | **1.00** | 1.34 | 42.74 | 17.99 | 5.89 |

| **SiHa** |  | | | | | | | | | |
| --- | --- | --- | --- | --- | --- | --- | --- | --- | --- | --- |
| Lane → | **1** | **2** | **3** | **4** | **5** | **6** | **7** | **8** | **9** | **10** |
| **p53** | **0.95** | 0.31 | 0.80 | 0.26 | 0.09 | 1.55 | 0.84 | 0.86 | 0.45 | 0.02 |
|  | **1.00** | 0.33 | 0.85 | 0.27 | 0.10 | 1.64 | 0.89 | 0.91 | 0.47 | 0.02 |

| **SiHa** |  | | | | | | | | | |
| --- | --- | --- | --- | --- | --- | --- | --- | --- | --- | --- |
| Lane → | **1** | **2** | **3** | **4** | **5** | **6** | **7** | **8** | **9** | **10** |
| **BID** | **0.89** | 0.88 | 0.74 | 0.74 | 0.75 | 0.81 | 0.86 | 1.10 | 1.13 | 0.94 |
|  | **1.00** | 0.99 | 0.83 | 0.83 | 0.84 | 0.91 | 0.96 | 1.23 | 1.26 | 1.06 |

| **SiHa** |  | | | | | | | | | |
| --- | --- | --- | --- | --- | --- | --- | --- | --- | --- | --- |
| Lane → | **1** | **2** | **3** | **4** | **5** | **6** | **7** | **8** | **9** | **10** |
| **t-BID** | 0.05 | 0.13 | 0.64 | 1.13 | 1.23 | **0.56** | 0.33 | 3.18 | 0.79 | 1.19 |
|  | 0.09 | 0.23 | 1.14 | 2.00 | 2.19 | **1.00** | 0.58 | 5.64 | 1.39 | 2.12 |

| **SiHa** |  | | | | | | | | | |
| --- | --- | --- | --- | --- | --- | --- | --- | --- | --- | --- |
| Lane → | **1** | **2** | **3** | **4** | **5** | **6** | **7** | **8** | **9** | **10** |
| **HPVE6** | **1.12** | 1.13 | 0.28 | 0.02 | 0.03 | 0.81 | 0.15 | 0.02 | 0.01 | 0.00 |
|  | **1.00** | 1.01 | 0.25 | 0.02 | 0.02 | 0.73 | 0.13 | 0.02 | 0.01 | 0.00 |

| **SiHa** |  | | | | | | | | | |
| --- | --- | --- | --- | --- | --- | --- | --- | --- | --- | --- |
| Lane → | **1** | **2** | **3** | **4** | **5** | **6** | **7** | **8** | **9** | **10** |
| **HPVE7** | **1.62** | 1.69 | 0.10 | 0.01 | 0.01 | 0.75 | 0.15 | 0.01 | 0.00 | 0.01 |
|  | **1.00** | 1.04 | 0.06 | 0.01 | 0.01 | 0.46 | 0.09 | 0.00 | 0.00 | 0.00 |

Figure 5A:

| **HeLa** |  | | | |
| --- | --- | --- | --- | --- |
| Lane → | **1** | **2** | **3** | **4** |
| **cl PARP** | 0.13 | **1.20** | 0.10 | 0.11 |
|  | 0.11 | **1.00** | 0.08 | 0.09 |

| **HeLa** |  | | | |
| --- | --- | --- | --- | --- |
| Lane → | **1** | **2** | **3** | **4** |
| **cl Caspase9** | 0.03 | **1.42** | 0.15 | 0.37 |
|  | 0.02 | **1.00** | 0.11 | 0.26 |

| **HeLa** |  | | | |
| --- | --- | --- | --- | --- |
| Lane → | **1** | **2** | **3** | **4** |
| **p53** | **1.18** | 2.34 | 0.03 | 0.02 |
|  | **1.00** | 1.98 | 0.02 | 0.02 |

| **HeLa** |  | | | |
| --- | --- | --- | --- | --- |
| Lane → | **1** | **2** | **3** | **4** |
| **P-p53**  **Ser5** | 0.02 | **1.30** | 0.01 | 0.01 |
|  | 0.02 | **1.00** | 0.01 | 0.00 |

| **HeLa** |  | | | |
| --- | --- | --- | --- | --- |
| Lane → | **1** | **2** | **3** | **4** |
| **Ac-p53**  **Lys382** | 0.09 | **1.64** | 0.02 | 0.02 |
|  | 0.06 | **1.00** | 0.01 | 0.01 |

| **HeLa** |  | | | |
| --- | --- | --- | --- | --- |
| Lane → | **1** | **2** | **3** | **4** |
| **BID** | **0.90** | 1.83 | 1.47 | 1.85 |
|  | **1.00** | 2.04 | 1.64 | 2.06 |

| **HeLa** |  | | | |
| --- | --- | --- | --- | --- |
| Lane → | **1** | **2** | **3** | **4** |
| **t-BID** | 0.14 | **1.64** | 0.25 | 0.57 |
|  | 0.09 | **1.00** | 0.15 | 0.34 |

Figure 5B HeLa:

| **HeLa** |  | | | |
| --- | --- | --- | --- | --- |
| Lane → | **1** | **2** | **3** | **4** |
| **cl PARP** | 0.10 | 0.17 | **1.63** | 0.56 |
|  | 0.06 | 0.10 | **1.00** | 0.34 |

| **HeLa** |  | | | |
| --- | --- | --- | --- | --- |
| Lane → | **1** | **2** | **3** | **4** |
| **cl Caspase9** | 0.00 | 0.03 | **0.98** | 0.37 |
|  | 0.00 | 0.03 | **1.00** | 0.38 |

| **HeLa** |  | | | |
| --- | --- | --- | --- | --- |
| Lane → | **1** | **2** | **3** | **4** |
| **p53** | **0.45** | 0.04 | 0.96 | 0.34 |
|  | **1.00** | 0.08 | 2.14 | 0.75 |

| **HeLa** |  | | | |
| --- | --- | --- | --- | --- |
| Lane → | **1** | **2** | **3** | **4** |
| **P-p53**  **Ser5** | 0.01 | 0.02 | **0.79** | 0.24 |
|  | 0.01 | 0.02 | **1.00** | 0.30 |

| **HeLa** |  | | | |
| --- | --- | --- | --- | --- |
| Lane → | **1** | **2** | **3** | **4** |
| **Ac-p53**  **Lys382** | 0.15 | 0.24 | **0.86** | 0.13 |
|  | 0.17 | 0.27 | **1.00** | 0.15 |

| **HeLa** |  | | | |
| --- | --- | --- | --- | --- |
| Lane → | **1** | **2** | **3** | **4** |
| **BID** | **0.96** | 1.33 | 1.14 | 1.02 |
|  | **1.00** | 1.38 | 1.18 | 1.06 |

| **HeLa** |  | | | |
| --- | --- | --- | --- | --- |
| Lane → | **1** | **2** | **3** | **4** |
| **t-BID** | 0.00 | 0.01 | **0.95** | 0.03 |
|  | 0.00 | 0.01 | **1.00** | 0.03 |

Figure 5B SiHa:

| **SiHa** |  | | | |
| --- | --- | --- | --- | --- |
| Lane → | **1** | **2** | **3** | **4** |
| **cl PARP** | 0.07 | 0.04 | **0.68** | 0.02 |
|  | 0.10 | 0.06 | **1.00** | 0.04 |

| **SiHa** |  | | | |
| --- | --- | --- | --- | --- |
| Lane → | **1** | **2** | **3** | **4** |
| **cl Caspase9** | 0.09 | 0.09 | **0.55** | 0.00 |
|  | 0.17 | 0.17 | **1.00** | 0.00 |

| **SiHa** |  | | | |
| --- | --- | --- | --- | --- |
| Lane → | **1** | **2** | **3** | **4** |
| **p53** | **0.55** | 0.16 | 0.85 | 0.03 |
|  | **1.00** | 0.30 | 1.55 | 0.06 |

| **SiHa** |  | | | |
| --- | --- | --- | --- | --- |
| Lane → | **1** | **2** | **3** | **4** |
| **P-p53**  **Ser5** | 0.06 | 0.01 | **1.13** | 0.01 |
|  | 0.05 | 0.01 | **1.00** | 0.01 |

| **SiHa** |  | | | |
| --- | --- | --- | --- | --- |
| Lane → | **1** | **2** | **3** | **4** |
| **Ac-p53**  **Lys382** | 0.35 | 0.22 | **1.19** | 0.21 |
|  | 0.29 | 0.18 | **1.00** | 0.18 |

| **SiHa** |  | | | |
| --- | --- | --- | --- | --- |
| Lane → | **1** | **2** | **3** | **4** |
| **BID** | **1.03** | 0.79 | 0.98 | 0.75 |
|  | **1.00** | 0.77 | 0.95 | 0.73 |

| **SiHa** |  | | | |
| --- | --- | --- | --- | --- |
| Lane → | **1** | **2** | **3** | **4** |
| **t-BID** | 0.08 | 0.05 | **1.06** | 0.13 |
|  | 0.08 | 0.05 | **1.00** | 0.12 |

Figure 6 HeLa:

| **HeLa** |  | | | | | | | | | | | |
| --- | --- | --- | --- | --- | --- | --- | --- | --- | --- | --- | --- | --- |
| Lane → | **1** | **2** | **3** | **4** | **5** | **6** | **7** | **8** | **9** | **10** | **11** | **12** |
| **cl PARP** | 0.01 | 0.06 | **0.04** | 1.15 | 0.01 | 0.01 | 0.01 | 0.25 | 0.01 | 0.01 | 0.01 | 0.39 |
|  | 0.14 | 1.32 | **1.00** | 25.96 | 0.31 | 0.20 | 0.16 | 5.63 | 0.16 | 0.19 | 0.13 | 8.79 |

| **HeLa** |  | | | | | | | | | | | |
| --- | --- | --- | --- | --- | --- | --- | --- | --- | --- | --- | --- | --- |
| Lane → | **1** | **2** | **3** | **4** | **5** | **6** | **7** | **8** | **9** | **10** | **11** | **12** |
| **cl Caspase9** | 0.01 | 0.11 | **0.17** | 1.00 | 0.01 | 0.02 | 0.04 | 0.26 | 0.00 | 0.01 | 0.01 | 0.57 |
|  | 0.04 | 0.65 | **1.00** | 5.83 | 0.08 | 0.10 | 0.23 | 1.52 | 0.03 | 0.05 | 0.06 | 3.33 |

| **HeLa** |  | | | | | | | | | | | |
| --- | --- | --- | --- | --- | --- | --- | --- | --- | --- | --- | --- | --- |
| Lane → | **1** | **2** | **3** | **4** | **5** | **6** | **7** | **8** | **9** | **10** | **11** | **12** |
| **p53** | **0.51** | 1.18 | 1.10 | 0.77 | 0.03 | 0.00 | 0.04 | 0.02 | 0.37 | 0.36 | 0.56 | 1.20 |
|  | **1.00** | 2.30 | 2.13 | 1.49 | 0.05 | 0.01 | 0.07 | 0.04 | 0.72 | 0.70 | 1.09 | 2.33 |

| **HeLa** |  | | | | | | | | | | | |
| --- | --- | --- | --- | --- | --- | --- | --- | --- | --- | --- | --- | --- |
| Lane → | **1** | **2** | **3** | **4** | **5** | **6** | **7** | **8** | **9** | **10** | **11** | **12** |
| **BID** | **1.66** | 1.71 | 1.93 | 1.34 | 1.52 | 1.11 | 1.24 | 1.09 | 0.14 | 0.20 | 0.14 | 0.20 |
|  | **1.00** | 1.03 | 1.16 | 0.80 | 0.91 | 0.67 | 0.74 | 0.66 | 0.09 | 0.12 | 0.08 | 0.12 |

| **HeLa** |  | | | | | | | | | | | |
| --- | --- | --- | --- | --- | --- | --- | --- | --- | --- | --- | --- | --- |
| Lane → | **1** | **2** | **3** | **4** | **5** | **6** | **7** | **8** | **9** | **10** | **11** | **12** |
| **t-BID** | 0.01 | 0.04 | **0.07** | 1.46 | 0.04 | 0.05 | 0.14 | 0.18 | 0.00 | 0.03 | 0.03 | 0.01 |
|  | 0.08 | 0.61 | **1.00** | 21.82 | 0.66 | 0.69 | 2.11 | 2.65 | 0.02 | 0.47 | 0.46 | 0.17 |

Figure 6 SiHa:

| **SiHa** |  | | | | | | | | | | | |
| --- | --- | --- | --- | --- | --- | --- | --- | --- | --- | --- | --- | --- |
| Lane → | **1** | **2** | **3** | **4** | **5** | **6** | **7** | **8** | **9** | **10** | **11** | **12** |
| **cl PARP** | 0.02 | 0.03 | **0.59** | 0.97 | 0.02 | 0.02 | 0.16 | 0.12 | 0.01 | 0.01 | 0.01 | 0.06 |
|  | 0.03 | 0.05 | **1.00** | 1.65 | 0.03 | 0.03 | 0.28 | 0.21 | 0.02 | 0.02 | 0.02 | 0.10 |

| **SiHa** |  | | | | | | | | | | | |
| --- | --- | --- | --- | --- | --- | --- | --- | --- | --- | --- | --- | --- |
| Lane → | **1** | **2** | **3** | **4** | **5** | **6** | **7** | **8** | **9** | **10** | **11** | **12** |
| **cl Caspase9** | 0.05 | 0.07 | **0.50** | 1.24 | 0.05 | 0.08 | 0.13 | 0.11 | 0.05 | 0.07 | 0.03 | 0.08 |
|  | 0.10 | 0.14 | **1.00** | 2.51 | 0.10 | 0.16 | 0.27 | 0.22 | 0.11 | 0.14 | 0.06 | 0.17 |

| **SiHa** |  | | | | | | | | | | | |
| --- | --- | --- | --- | --- | --- | --- | --- | --- | --- | --- | --- | --- |
| Lane → | **1** | **2** | **3** | **4** | **5** | **6** | **7** | **8** | **9** | **10** | **11** | **12** |
| **p53** | **0.84** | 0.73 | 1.72 | 0.60 | 0.04 | 0.01 | 0.03 | 0.03 | 0.98 | 1.11 | 1.30 | 0.54 |
|  | **1.00** | 0.87 | 2.05 | 0.71 | 0.05 | 0.01 | 0.04 | 0.04 | 1.16 | 1.32 | 1.55 | 0.64 |

| **SiHa** |  | | | | | | | | | | | |
| --- | --- | --- | --- | --- | --- | --- | --- | --- | --- | --- | --- | --- |
| Lane → | **1** | **2** | **3** | **4** | **5** | **6** | **7** | **8** | **9** | **10** | **11** | **12** |
| **BID** | **1.49** | 1.46 | 1.47 | 1.11 | 1.17 | 1.00 | 1.13 | 0.87 | 0.19 | 0.15 | 0.09 | 0.07 |
|  | **1.00** | 0.98 | 0.98 | 0.74 | 0.79 | 0.67 | 0.76 | 0.58 | 0.12 | 0.10 | 0.06 | 0.05 |

| **SiHa** |  | | | | | | | | | | | |
| --- | --- | --- | --- | --- | --- | --- | --- | --- | --- | --- | --- | --- |
| Lane → | **1** | **2** | **3** | **4** | **5** | **6** | **7** | **8** | **9** | **10** | **11** | **12** |
| **t-BID** | 0.01 | 0.07 | **0.55** | 1.79 | 0.07 | 0.07 | 0.13 | 0.17 | 0.03 | 0.02 | 0.05 | 0.04 |
|  | 0.02 | 0.14 | **1.00** | 3.26 | 0.14 | 0.13 | 0.24 | 0.31 | 0.06 | 0.04 | 0.09 | 0.07 |

Figure S1B SiHa:

| **SiHa** |  | | | | | | | | | | | |
| --- | --- | --- | --- | --- | --- | --- | --- | --- | --- | --- | --- | --- |
| Lane → | **1** | **2** | **3** | **4** | **5** | **6** | **7** | **8** | **9** | **10** | **11** | **12** |
| **p53** | **0.15** | 0.18 | 0.02 | 0.03 | 0.79 | 1.09 | 0.14 | 0.26 | 1.06 | 0.97 | 0.12 | 0.17 |
|  | **1.00** | 1.20 | 0.13 | 0.20 | 5.43 | 7.51 | 0.93 | 1.75 | 7.26 | 6.65 | 0.79 | 1.17 |

| **SiHa** |  | | | | | | | | | | | |
| --- | --- | --- | --- | --- | --- | --- | --- | --- | --- | --- | --- | --- |
| Lane → | **1** | **2** | **3** | **4** | **5** | **6** | **7** | **8** | **9** | **10** | **11** | **12** |
| **p21** | **0.03** | 0.01 | 0.09 | 0.02 | 0.81 | 1.45 | 0.22 | 0.07 | 0.87 | 0.69 | 0.01 | 0.00 |
|  | **1.00** | 0.36 | 2.54 | 0.49 | 23.90 | 43.02 | 6.55 | 2.04 | 25.91 | 20.40 | 0.38 | 0.00 |

| **SiHa** |  | | | | | | | | | | | |
| --- | --- | --- | --- | --- | --- | --- | --- | --- | --- | --- | --- | --- |
| Lane → | **1** | **2** | **3** | **4** | **5** | **6** | **7** | **8** | **9** | **10** | **11** | **12** |
| **E6AP** | **0.70** | 0.60 | 0.70 | 0.61 | 0.05 | 0.02 | 0.11 | 0.18 | 0.83 | 0.60 | 0.78 | 0.85 |
|  | **1.00** | 0.86 | 1.00 | 0.87 | 0.07 | 0.03 | 0.15 | 0.25 | 1.18 | 0.85 | 1.11 | 1.21 |

| **SiHa** |  | | | | | | | | | | | |
| --- | --- | --- | --- | --- | --- | --- | --- | --- | --- | --- | --- | --- |
| Lane → | **1** | **2** | **3** | **4** | **5** | **6** | **7** | **8** | **9** | **10** | **11** | **12** |
| **HPVE6** | **1.26** | 0.78 | 0.32 | 0.05 | 0.01 | 0.02 | 0.10 | 0.01 | 0.02 | 0.00 | 0.00 | 0.00 |
|  | **1.00** | 0.62 | 0.26 | 0.04 | 0.00 | 0.02 | 0.08 | 0.00 | 0.02 | 0.00 | 0.00 | 0.00 |

| **SiHa** |  | | | | | | | | | | | |
| --- | --- | --- | --- | --- | --- | --- | --- | --- | --- | --- | --- | --- |
| Lane → | **1** | **2** | **3** | **4** | **5** | **6** | **7** | **8** | **9** | **10** | **11** | **12** |
| **HPVE7** | **1.39** | 1.20 | 0.06 | 0.04 | 1.15 | 0.81 | 0.07 | 0.02 | 0.14 | 0.05 | 0.00 | 0.00 |
|  | **1.00** | 0.86 | 0.05 | 0.03 | 0.83 | 0.59 | 0.05 | 0.02 | 0.10 | 0.03 | 0.00 | 0.00 |

Figure S1C U2OS:

| **U2OS** |  | | |
| --- | --- | --- | --- |
| Lane → | **1** | **2** | **3** |
| **p53** | **0.68** | 0.48 | 0.07 |
|  | **1.00** | 0.71 | 0.10 |

| **U2OS** |  | | |
| --- | --- | --- | --- |
| Lane → | **1** | **2** | **3** |
| **p21** | **0.88** | 0.05 | 0.03 |
|  | **1.00** | 0.06 | 0.04 |

Figure S1D U2OS:

| **U2OS** |  | | | |
| --- | --- | --- | --- | --- |
| Lane → | **1** | **2** | **3** | **4** |
| **p53** | **0.32** | 0.24 | 0.91 | 0.03 |
|  | **1.00** | 0.75 | 2.81 | 0.09 |

| **U2OS** |  | | | |
| --- | --- | --- | --- | --- |
| Lane → | **1** | **2** | **3** | **4** |
| **p21** | **0.60** | 0.16 | 1.68 | 0.08 |
|  | **1.00** | 0.26 | 2.79 | 0.14 |

Figure S1D HCT116:

| **HCT116** |  | | | |
| --- | --- | --- | --- | --- |
| Lane → | **1** | **2** | **3** | **4** |
| **p53** | **0.21** | 0.14 | 1.22 | 0.42 |
|  | **1.00** | 0.66 | 5.86 | 2.03 |

| **HCT116** |  | | | |
| --- | --- | --- | --- | --- |
| Lane → | **1** | **2** | **3** | **4** |
| **p21** | **0.71** | 0.14 | 1.86 | 0.08 |
|  | **1.00** | 0.19 | 2.62 | 0.11 |

Figure S4:

| **HeLa** |  | | | | | | | | |
| --- | --- | --- | --- | --- | --- | --- | --- | --- | --- |
| Lane → | **1** | **2** | **3** | **4** | **5** | **6** | **7** | **8** | **9** |
| **HPVE6** | **1.04** | 1.20 | 0.99 | 0.04 | 0.00 | 0.01 | 0.76 | 0.69 | 0.59 |
|  | **1.00** | 1.16 | 0.96 | 0.04 | 0.00 | 0.00 | 0.73 | 0.67 | 0.57 |

| **HeLa** |  | | | | | | | | |
| --- | --- | --- | --- | --- | --- | --- | --- | --- | --- |
| Lane → | **1** | **2** | **3** | **4** | **5** | **6** | **7** | **8** | **9** |
| **HPVE7** | **0.64** | 1.11 | 0.91 | 0.26 | 0.14 | 0.15 | 1.05 | 1.06 | 0.92 |
|  | **1.00** | 1.74 | 1.42 | 0.40 | 0.22 | 0.24 | 1.64 | 1.65 | 1.44 |

Figure S9 HeLa:

| **HeLa** |  | | | | | | | | | | | |
| --- | --- | --- | --- | --- | --- | --- | --- | --- | --- | --- | --- | --- |
| Lane → | **1** | **2** | **3** | **4** | **5** | **6** | **7** | **8** | **9** | **10** | **11** | **12** |
| **p53** | **0.98** | 0.85 | 0.76 | 0.68 | 0.57 | 0.82 | 0.18 | 1.69 | 2.02 | 0.10 | 1.18 | 0.98 |
|  | **1.00** | 0.87 | 0.78 | 0.69 | 0.58 | 0.84 | 0.18 | 1.73 | 2.07 | 0.10 | 1.20 | 1.00 |

| **HeLa** |  | | | | | | | | | | | |
| --- | --- | --- | --- | --- | --- | --- | --- | --- | --- | --- | --- | --- |
| Lane → | **1** | **2** | **3** | **4** | **5** | **6** | **7** | **8** | **9** | **10** | **11** | **12** |
| **P-p53**  **Ser15** | **0.16** | 0.13 | 0.15 | 0.14 | 0.02 | 0.14 | 0.20 | 0.79 | 1.15 | 0.00 | 0.37 | 0.32 |
|  | **1.00** | 0.80 | 0.92 | 0.87 | 0.15 | 0.84 | 1.25 | 4.88 | 7.08 | 0.03 | 2.30 | 1.95 |

| **HeLa** |  | | | | | | | | | | | |
| --- | --- | --- | --- | --- | --- | --- | --- | --- | --- | --- | --- | --- |
| Lane → | **1** | **2** | **3** | **4** | **5** | **6** | **7** | **8** | **9** | **10** | **11** | **12** |
| **Ac-p53**  **Lys382** | **0.50** | 0.10 | 0.17 | 0.17 | 0.05 | 0.11 | 0.01 | 0.77 | 0.79 | 0.12 | 0.60 | 0.21 |
|  | **1.00** | 0.21 | 0.33 | 0.35 | 0.11 | 0.22 | 0.02 | 1.54 | 1.58 | 0.24 | 1.20 | 0.42 |

| **HeLa** |  | | | | | | | | | | | |
| --- | --- | --- | --- | --- | --- | --- | --- | --- | --- | --- | --- | --- |
| Lane → | **1** | **2** | **3** | **4** | **5** | **6** | **7** | **8** | **9** | **10** | **11** | **12** |
| **HPVE6** | **1.87** | 1.72 | 1.87 | 1.32 | 1.07 | 1.38 | 0.29 | 0.01 | 0.00 | 0.16 | 0.00 | 0.00 |
|  | **1.00** | 0.92 | 1.00 | 0.71 | 0.57 | 0.74 | 0.16 | 0.01 | 0.00 | 0.09 | 0.00 | 0.00 |

| **HeLa** |  | | | | | | | | | | | |
| --- | --- | --- | --- | --- | --- | --- | --- | --- | --- | --- | --- | --- |
| Lane → | **1** | **2** | **3** | **4** | **5** | **6** | **7** | **8** | **9** | **10** | **11** | **12** |
| **HPVE7** | **1.39** | 1.48 | 1.78 | 0.82 | 1.14 | 1.40 | 0.19 | 0.00 | 0.03 | 0.26 | 0.05 | 0.03 |
|  | **1.00** | 1.07 | 1.28 | 0.59 | 0.83 | 1.01 | 0.14 | 0.00 | 0.02 | 0.19 | 0.04 | 0.02 |

Figure S9 SiHa:

| **SiHa** |  | | | | | | | | | | | |
| --- | --- | --- | --- | --- | --- | --- | --- | --- | --- | --- | --- | --- |
| Lane → | **1** | **2** | **3** | **4** | **5** | **6** | **7** | **8** | **9** | **10** | **11** | **12** |
| **p53** | **0.70** | 0.76 | 0.80 | 0.40 | 0.55 | 0.66 | 0.27 | 0.31 | 0.85 | 0.06 | 0.23 | 0.28 |
|  | **1.00** | 1.07 | 1.14 | 0.58 | 0.78 | 0.94 | 0.38 | 0.43 | 1.21 | 0.09 | 0.32 | 0.40 |

| **SiHa** |  | | | | | | | | | | | |
| --- | --- | --- | --- | --- | --- | --- | --- | --- | --- | --- | --- | --- |
| Lane → | **1** | **2** | **3** | **4** | **5** | **6** | **7** | **8** | **9** | **10** | **11** | **12** |
| **P-p53**  **Ser15** | 0.01 | 0.04 | **0.22** | 0.04 | 0.02 | 0.11 | 0.01 | 0.03 | 1.41 | 0.00 | 0.01 | 0.06 |
|  | 0.05 | 0.20 | **1.00** | 0.19 | 0.09 | 0.49 | 0.03 | 0.14 | 6.46 | 0.02 | 0.03 | 0.29 |

| **SiHa** |  | | | | | | | | | | | |
| --- | --- | --- | --- | --- | --- | --- | --- | --- | --- | --- | --- | --- |
| Lane → | **1** | **2** | **3** | **4** | **5** | **6** | **7** | **8** | **9** | **10** | **11** | **12** |
| **Ac-p53**  **Lys382** | 0.14 | 0.16 | **0.29** | 0.16 | 0.05 | 0.09 | 0.00 | 0.05 | 0.94 | 0.05 | 0.05 | 0.02 |
|  | 0.49 | 0.55 | **1.00** | 0.55 | 0.17 | 0.32 | 0.01 | 0.17 | 3.24 | 0.19 | 0.17 | 0.08 |

| **SiHa** |  | | | | | | | | | | | |
| --- | --- | --- | --- | --- | --- | --- | --- | --- | --- | --- | --- | --- |
| Lane → | **1** | **2** | **3** | **4** | **5** | **6** | **7** | **8** | **9** | **10** | **11** | **12** |
| **HPVE6** | **0.65** | 0.64 | 0.85 | 0.89 | 0.66 | 0.28 | 0.37 | 0.06 | 0.01 | 0.12 | 0.01 | 0.00 |
|  | **1.00** | 0.98 | 1.30 | 1.37 | 1.01 | 0.43 | 0.57 | 0.09 | 0.02 | 0.19 | 0.01 | 0.00 |

| **SiHa** |  | | | | | | | | | | | |
| --- | --- | --- | --- | --- | --- | --- | --- | --- | --- | --- | --- | --- |
| Lane → | **1** | **2** | **3** | **4** | **5** | **6** | **7** | **8** | **9** | **10** | **11** | **12** |
| **HPVE7** | **1.03** | 0.87 | 0.83 | 1.18 | 1.03 | 0.22 | 0.21 | 0.06 | 0.01 | 0.02 | 0.04 | 0.03 |
|  | **1.00** | 0.85 | 0.81 | 1.15 | 1.00 | 0.21 | 0.21 | 0.06 | 0.01 | 0.02 | 0.04 | 0.03 |

Figure S10A:

| **HeLa** |  |  |  |  |  |  |
| --- | --- | --- | --- | --- | --- | --- |
| Lane → | **1** | **2** | **3** | **4** | **5** | **6** |
| **cl PARP** | 0.08 | **1.87** | 0.15 | 1.25 | 0.10 | 0.19 |
|  | 0.04 | **1.00** | 0.08 | 0.67 | 0.06 | 0.10 |

| **HeLa** |  |  |  |  |  |  |
| --- | --- | --- | --- | --- | --- | --- |
| Lane → | **1** | **2** | **3** | **4** | **5** | **6** |
| **cl Caspase9** | 0.02 | **0.89** | 0.05 | 0.99 | 0.04 | 0.12 |
|  | 0.02 | **1.00** | 0.06 | 1.11 | 0.05 | 0.14 |

| **HeLa** |  |  |  |  |  |  |
| --- | --- | --- | --- | --- | --- | --- |
| Lane → | **1** | **2** | **3** | **4** | **5** | **6** |
| **p53** | **0.41** | 1.00 | 0.56 | 1.16 | 0.46 | 0.41 |
|  | **1.00** | 2.45 | 1.36 | 2.83 | 1.13 | 1.00 |

| **HeLa** |  |  |  |  |  |  |
| --- | --- | --- | --- | --- | --- | --- |
| Lane → | **1** | **2** | **3** | **4** | **5** | **6** |
| **HPVE6** | **1.24** | 0.32 | 1.03 | 0.24 | 0.05 | 0.01 |
|  | **1.00** | 0.26 | 0.83 | 0.20 | 0.04 | 0.01 |

| **HeLa** |  |  |  |  |  |  |
| --- | --- | --- | --- | --- | --- | --- |
| Lane → | **1** | **2** | **3** | **4** | **5** | **6** |
| **HPVE7** | **0.94** | 0.16 | 0.85 | 0.21 | 0.25 | 0.20 |
|  | **1.00** | 0.17 | 0.90 | 0.23 | 0.26 | 0.21 |

Figure S10B:

| **HeLa** |  |  |  |  |  |  |
| --- | --- | --- | --- | --- | --- | --- |
| Lane → | **1** | **2** | **3** | **4** | **5** | **6** |
| **cl PARP** | 0.01 | **0.10** | 0.48 | 0.60 | 0.33 | 1.08 |
|  | 0.07 | **1.00** | 4.71 | 5.84 | 3.17 | 10.48 |

| **HeLa** |  |  |  |  |  |  |
| --- | --- | --- | --- | --- | --- | --- |
| Lane → | **1** | **2** | **3** | **4** | **5** | **6** |
| **cl Caspase9** | 0.09 | **0.41** | 0.81 | 0.92 | 0.36 | 1.10 |
|  | 0.22 | **1.00** | 1.98 | 2.24 | 0.89 | 2.69 |

| **HeLa** |  |  |  |  |  |  |
| --- | --- | --- | --- | --- | --- | --- |
| Lane → | **1** | **2** | **3** | **4** | **5** | **6** |
| **p53** | **0.25** | 0.81 | 0.26 | 0.84 | 1.11 | 0.72 |
|  | **1.00** | 3.29 | 1.04 | 3.43 | 4.49 | 2.93 |

| **HeLa** |  |  |  |  |  |  |
| --- | --- | --- | --- | --- | --- | --- |
| Lane → | **1** | **2** | **3** | **4** | **5** | **6** |
| **HPVE6** | **0.79** | 0.86 | 0.62 | 0.48 | 0.05 | 0.01 |
|  | **1.00** | 1.09 | 0.79 | 0.61 | 0.06 | 0.02 |

| **HeLa** |  |  |  |  |  |  |
| --- | --- | --- | --- | --- | --- | --- |
| Lane → | **1** | **2** | **3** | **4** | **5** | **6** |
| **HPVE7** | **1.30** | 1.09 | 1.32 | 1.06 | 0.10 | 0.01 |
|  | **1.00** | 0.83 | 1.01 | 0.81 | 0.07 | 0.01 |

Figure S11:

| **SiHa** |  | | | |
| --- | --- | --- | --- | --- |
| Lane → | **1** | **2** | **3** | **4** |
| **cl PARP** | 0.01 | 0.02 | **0.64** | 0.24 |
|  | 0.01 | 0.03 | **1.00** | 0.38 |

| **SiHa** |  | | | |
| --- | --- | --- | --- | --- |
| Lane → | **1** | **2** | **3** | **4** |
| **cl Caspase9** | 0.00 | 0.01 | **0.64** | 0.01 |
|  | 0.00 | 0.02 | **1.00** | 0.01 |

| **SiHa** |  | | | |
| --- | --- | --- | --- | --- |
| Lane → | **1** | **2** | **3** | **4** |
| **p53** | **0.46** | 0.07 | 1.68 | 0.10 |
|  | **1.00** | 0.15 | 3.67 | 0.22 |

| **SiHa** |  | | | |
| --- | --- | --- | --- | --- |
| Lane → | **1** | **2** | **3** | **4** |
| **P-p53 Ser15** | 0.03 | 0.03 | **1.32** | 0.02 |
|  | 0.03 | 0.03 | **1.00** | 0.02 |

| **SiHa** |  | | | |
| --- | --- | --- | --- | --- |
| Lane → | **1** | **2** | **3** | **4** |
| **Ac-p53 Lys382** | 0.05 | 0.02 | **0.94** | 0.00 |
|  | 0.05 | 0.02 | **1.00** | 0.00 |

| **SiHa** |  | | | |
| --- | --- | --- | --- | --- |
| Lane → | **1** | **2** | **3** | **4** |
| **BID** | **1.40** | 1.40 | 1.33 | 1.00 |
|  | **1.00** | 1.00 | 0.95 | 0.72 |

| **SiHa** |  | | | |
| --- | --- | --- | --- | --- |
| Lane → | **1** | **2** | **3** | **4** |
| **t-BID** | 0.26 | 0.29 | **1.54** | 0.14 |
|  | 0.17 | 0.19 | **1.00** | 0.09 |

Figure S12:

| **HeLa** |  | | | | | | | |
| --- | --- | --- | --- | --- | --- | --- | --- | --- |
| Lane → | **1** | **2** | **3** | **4** | **5** | **6** | **7** | **8** |
| **cl PARP** | 0.03 | 0.03 | **0.05** | 1.14 | 0.05 | 0.05 | 0.05 | 0.15 |
|  | 0.67 | 0.62 | **1.00** | 24.49 | 0.98 | 0.97 | 1.03 | 3.30 |

| **HeLa** |  | | | | | | | |
| --- | --- | --- | --- | --- | --- | --- | --- | --- |
| Lane → | **1** | **2** | **3** | **4** | **5** | **6** | **7** | **8** |
| **cl Caspase9** | 0.01 | 0.04 | **0.17** | 0.73 | 0.01 | 0.01 | 0.04 | 0.49 |
|  | 0.06 | 0.23 | **1.00** | 4.16 | 0.07 | 0.07 | 0.21 | 2.83 |

| **HeLa** |  | | | | | | | |
| --- | --- | --- | --- | --- | --- | --- | --- | --- |
| Lane → | **1** | **2** | **3** | **4** | **5** | **6** | **7** | **8** |
| **p53** | **0.45** | 0.35 | 1.06 | 0.93 | 0.08 | 0.03 | 0.15 | 0.24 |
|  | **1.00** | 0.78 | 2.36 | 2.06 | 0.17 | 0.07 | 0.33 | 0.54 |

| **HeLa** |  | | | | | | | |
| --- | --- | --- | --- | --- | --- | --- | --- | --- |
| Lane → | **1** | **2** | **3** | **4** | **5** | **6** | **7** | **8** |
| **BID** | **0.59** | 0.82 | 0.92 | 0.84 | 0.84 | 0.85 | 0.91 | 1.07 |
|  | **1.00** | 1.40 | 1.57 | 1.43 | 1.43 | 1.45 | 1.54 | 1.82 |

| **HeLa** |  | | | | | | | |
| --- | --- | --- | --- | --- | --- | --- | --- | --- |
| Lane → | **1** | **2** | **3** | **4** | **5** | **6** | **7** | **8** |
| **t-BID** | 0.20 | 0.37 | **0.59** | 1.19 | 0.33 | 0.25 | 0.12 | 0.31 |
|  | 0.34 | 0.63 | **1.00** | 2.02 | 0.56 | 0.42 | 0.21 | 0.53 |

Figure S15 HeLa:

| **HeLa** |  | | | |
| --- | --- | --- | --- | --- |
| Lane → | **1** | **2** | **3** | **4** |
| **cl PARP** | 0.04 | 0.06 | **1.13** | 1.72 |
|  | 0.03 | 0.05 | **1.00** | 1.52 |

| **HeLa** |  | | | |
| --- | --- | --- | --- | --- |
| Lane → | **1** | **2** | **3** | **4** |
| **cl Caspase9** | 0.01 | 0.01 | **0.53** | 0.88 |
|  | 0.03 | 0.01 | **1.00** | 1.68 |

| **HeLa** |  | | | |
| --- | --- | --- | --- | --- |
| Lane → | **1** | **2** | **3** | **4** |
| **p53** | **0.11** | 0.20 | 1.16 | 1.16 |
|  | **1.00** | 1.75 | 10.13 | 10.05 |

| **HeLa** |  | | | |
| --- | --- | --- | --- | --- |
| Lane → | **1** | **2** | **3** | **4** |
| **HPVE6** | **1.17** | 1.75 | 0.13 | 0.03 |
|  | **1.00** | 1.50 | 0.11 | 0.03 |

| **HeLa** |  | | | |
| --- | --- | --- | --- | --- |
| Lane → | **1** | **2** | **3** | **4** |
| **HPVE7** | **1.03** | 1.56 | 0.42 | 0.17 |
|  | **1.00** | 1.52 | 0.41 | 0.16 |

Figure S15 SiHa:

| **SiHa** |  | | | |
| --- | --- | --- | --- | --- |
| Lane → | **1** | **2** | **3** | **4** |
| **cl PARP** | 0.02 | 0.04 | **1.74** | 1.37 |
|  | 0.01 | 0.02 | **1.00** | 0.79 |

| **SiHa** |  | | | |
| --- | --- | --- | --- | --- |
| Lane → | **1** | **2** | **3** | **4** |
| **cl Caspase9** | 0.01 | 0.03 | **1.05** | 0.91 |
|  | 0.01 | 0.03 | **1.00** | 0.86 |

| **SiHa** |  | | | |
| --- | --- | --- | --- | --- |
| Lane → | **1** | **2** | **3** | **4** |
| **p53** | **0.05** | 0.07 | 0.96 | 0.87 |
|  | **1.00** | 1.43 | 19.88 | 17.99 |

| **SiHa** |  | | | |
| --- | --- | --- | --- | --- |
| Lane → | **1** | **2** | **3** | **4** |
| **HPVE6** | **1.19** | 1.08 | 0.26 | 0.21 |
|  | **1.00** | 0.91 | 0.22 | 0.18 |

| **SiHa** |  | | | |
| --- | --- | --- | --- | --- |
| Lane → | **1** | **2** | **3** | **4** |
| **HPVE7** | **1.67** | 2.26 | 0.05 | 0.03 |
|  | **1.00** | 1.35 | 0.03 | 0.02 |
